# Non-Targeted Metabolomic Analysis of *Arabidopsis thaliana* (L.) Heynh: Metabolic Adaptive Responses to Stress Caused by N Starvation

**DOI:** 10.3390/metabo13091021

**Published:** 2023-09-18

**Authors:** Jorge David Cadena-Zamudio, Juan Luis Monribot-Villanueva, Claudia-Anahí Pérez-Torres, Fulgencio Alatorre-Cobos, José Antonio Guerrero-Analco, Enrique Ibarra-Laclette

**Affiliations:** 1 Red de Estudios Moleculares Avanzados (REMAV), Instituto de Ecología, A.C. (INECOL), Xalapa 91073, Veracruz, Mexico; cadenazamudioj@gmail.com (J.D.C.-Z.); juan.monribot@inecol.mx (J.L.M.-V.); claudia.perez@inecol.mx (C.-A.P.-T.); joseantonio.guerrero@inecol.mx (J.A.G.-A.); 2 Consejo Nacional de Ciencia y Tecnología, Unidad de Bioquímica y Biología Molecular de Plantas, Merida 97205, Yucatan, Mexico; fulgencio.alatorre@cicy.mx; 3 Centro de Investigación Científica de Yucatán (CICY), Unidad de Biotecnología, Merida 97205, Yucatan, Mexico

**Keywords:** *Arabidopsis thaliana*, non-targeted metabolomics, abiotic stress, metabolic plasticity

## Abstract

As sessile organisms, plants develop the ability to respond and survive in changing environments. Such adaptive responses maximize phenotypic and metabolic fitness, allowing plants to adjust their growth and development. In this study, we analyzed the metabolic plasticity of *Arabidopsis thaliana* in response to nitrate deprivation by untargeted metabolomic analysis and using wild-type (WT) genotypes and the loss-of-function *nia1*/*nia2* double mutant. Secondary metabolites were identified using seedlings grown on a hydroponic system supplemented with optimal or limiting concentrations of N (4 or 0.2 mM, respectively) and harvested at 15 and 30 days of age. Then, spectral libraries generated from shoots and roots in both ionization modes (ESI +/−) were compared. Totals of 3407 and 4521 spectral signals (m/z_rt) were obtained in the ESI^+^ and ESI^−^ modes, respectively. Of these, approximately 50 and 65% were identified as differentially synthetized/accumulated. This led to the presumptive identification of 735 KEGG codes (metabolites) belonging to 79 metabolic pathways. The metabolic responses in the shoots and roots of WT genotypes at 4 mM of N favor the synthesis/accumulation of metabolites strongly related to growth. In contrast, for the *nia1*/*nia2* double mutant (similar as the WT genotype at 0.2 mM N), metabolites identified as differentially synthetized/accumulated help cope with stress, regulating oxidative stress and preventing programmed cell death, meaning that metabolic responses under N starvation compromise growth to prioritize a defensive response.

## 1. Introduction

As sessile organisms, plants exist in a continuous dynamic with their environment. Their survival and success largely depend on their ability to adapt and respond to various environmental conditions [1]. A significant manifestation of this adaptation is phenotypic plasticity, which is the capacity of a single genotype to exhibit a range of phenotypes in response to environmental variation. This plasticity enables plants to cope with changing environments by altering their growth and development [2]. For example, N (an essential macronutrient) starvation, among other physiological and phenotypical traits, affects growth and plant development, root architecture, and chlorophyll synthesis [3,4,5].

Beyond the visual and structural changes in phenotypic plasticity, a more subtle but equally vital mechanism of the adaptation of plants is represented by their metabolic processes [6]. Metabolic plasticity refers to the modulation of metabolic pathways and shifts in metabolite composition that allow an organism to optimize its physiological and biochemical functions under varying environmental conditions. In plants, metabolic plasticity is an important mechanism to cope with environmental stresses, augmenting their survival capacity in challenging habitats [7].

Abiotic stress, which encompasses non-living environmental factors such as drought, extreme temperatures, salinity, and nutrient deficiency, poses significant challenges to plant survival and productivity [8,9]. Plants respond to abiotic stress through many physiological, biochemical, and molecular changes, including alterations in their metabolic networks. Consequently, abiotic stress can significantly impact the production and accumulation of metabolites in plants, profoundly affecting their growth, development, and overall fitness [9,10,11]. Among the broad array of changes, secondary metabolites (SMes) have been recognized for their critical roles in plant adaptation to environmental stress. They are often produced and accumulated in response to abiotic stress, acting as protective compounds that help plants defend against damage caused by stress conditions [10]. Such SMes, which include flavonoids, terpenoids, glucosinolates, and phenylpropanoids, among others, play integral roles in maintaining cellular homeostasis, quenching reactive oxygen species, and mitigating oxidative stress, thereby promoting plant survival under stress conditions [12,13]. In order to delve into the complex interplay between plant phenotypic and metabolic plasticity, abiotic stress, and the role of SMes, metabolomic studies arise as an optimal approach. Metabolomics represent an invaluable tool for the study of such phenomena through the comprehensive analysis of metabolites in biological systems. By simultaneously measuring thousands of metabolites, metabolomics allows for the detection of global metabolic changes in response to environmental stimuli, providing insights into the adaptive responses of plants [14,15,16]. In particular, non-targeted metabolomics represent a powerful tool for a holistic understanding of plant metabolisms under varying conditions. By detecting as many metabolites as possible without a predefined target list, this approach enables a broad-spectrum analysis of the plant metabolome, thereby capturing the full complexity of metabolic responses [14,16]. With this powerful tool, we can delve into the intricate network of plant metabolic and phenotypic responses to abiotic stress and the role of SMes therein [14]. In summary, understanding the mechanisms underlying the metabolic plasticity of plants in response to abiotic stress, specifically the role of secondary metabolites, is essential to improve our understanding of plant resilience. Therefore, in this work, we analyze the metabolic responses of *A. thaliana* within a temporal kinetic framework wherein contrasting nitrate concentrations (4 mM as a control and 0.2 mM representing severe stress) are employed across two distinct genotypes (wild-type (WT) and loss-of-function double-mutant *nia1*/*nia2*) characterized by an inability to reduce nitrates independently of their availability. Our overarching aim is to elucidate the intricate interplay between nitrate availability conditions and the resultant modulation of the plant’s metabolomic composition, concurrently deciphering the responses exhibited by both genotypes. With this study, we seek to unravel the variegated landscape of metabolic plasticity in *A. thaliana*, with a particular emphasis on unveiling the metabolic responses incited by oscillating nitrate conditions while also delving into the distinctive roles enacted by specific secondary metabolites in ameliorating the perturbations stemming from nutritional stress.

## 2. Materials and Methods

### 2.1. Biological Material

The methodology of this study consists of two parts. The first part corresponds to the methods used to carry out the assembly of time-course experiments. The second part fits the process of injection and non-targeted metabolomic analysis. The wild-type (WT) *A. thaliana* ecotype Col-0 and loss-of-function *nia1*/*nia2* double mutant (unable to reduce nitrates [17]) were used in time course experiments. The latter was kindly donated by Dr. Randy Ortiz Castro, belonging to INECOL’s Advanced Molecular Studies Network (REMAV).

### 2.2. Disinfection of Arabidopsis Thaliana Seeds

To germinate the *A. thaliana* seeds, first, a washing and disinfection phase was carried out using 1 mL of 70% ethanol in 1.5 mL plastic tubes for 5 min with shaking. Subsequently, ethanol was decanted, and 1 mL of 20% chlorine solution was added to the tubes, which were shaken for 7 min. Finally, seeds were washed with sterile distilled water (at least five times) and stored with sterile distilled water at 4 °C for 48 h to synchronize germination.

### 2.3. Preparation of Murashige and Skoog (MS) Medium for Hydroponic Cultures

The preparation of the MS liquid medium [18] was carried out (Table S1) at a concentration of 0.1×, supplemented with 5 g L^−1^ of sucrose and vitamins (Table S2), adjusting the pH to 5.7 with KOH, and modifying only the concentration of ammonium nitrate (NH_4_NO_3_) and potassium nitrate (KNO_3_) to obtain concentrations of 4 mM (optimal level also named as control) and 0.2 mM (N-depletion or severe stress). Once the medium was made, it was sterilized by conventional autoclaving at 121 °C for 21 min.

### 2.4. Construction of Hydroponic Chambers

Hydroponic chambers were constructed using containers (and their lids) of 500 and 1000 mL capacity. In addition to the high-density polyethylene (HDPE) containers, lycra mesh and cotton were used. Subsequently, the assembly of the hydroponic chambers was carried out, following the methodology described by Alatorre-Cobos et al. [19].

### 2.5. Growth Conditions and Times-Sampling

The time course experiment was established using the seeds of the two genotypes of *A. thaliana* (WT and *nia1*/*nia2*). A growth chamber (Percival, Perry, IA, USA) with fluorescent light (100 μmol m^−2^·s^−1^), constant temperature (22 °C) and a photoperiod of 16 h light/8 h dark was used. Approximately 400 mL of liquid MS medium was added to each hydroponic chamber until the medium moistened the mesh. Once the above was done, the seeds were sown inside the hydroponic chambers on the surface of the mesh [19], and seedlings were grown inside the chamber in both contrasting conditions of nitrates—optimal (4 mM) and N-depletion (0.2 mM), respectively. Two sampling points were chosen to harvest the seedlings: 15 and 30 days after germination (dag). Concerning the experimental design, each hydroponic chamber contained up of *n* = 6 (seeds/seedlings), and enough hydroponic chambers were made to obtain at least 12 replicates for each genotype (WT and *nia1*/*nia2*) in both sampling points (15 and 30 dag) and at both contrasting conditions of nitrates (4 and 0.2 mM). This complex experimental design provides the quantity of biomass required for subsequent analyses (Figure S1).

### 2.6. Quantification of Phenotypic Traits

The net biomass obtained from timecourse experiments was used to carry out the photo documentation, as well as the quantification of the fresh weight of the rosette and root and the measurement of the root length and the diameter of the rosette independently. This was done for each of the six plants contained within each hydroponic chamber, in each batch, belonging to the different times (15 or 30 dag), nitrate concentrations (4 or 0.2 mM), and corresponding genotypes (WT and *nia1*/*nia2*). Once the above was done, the total biomass was fractionated in order to obtain the aerial biomass (rosette) and the root biomass separately, and thus carry out the subsequent metabolite extraction and identification.

### 2.7. Methanolic Extracts

The biomass collected from the different treatments was lyophilized using LABCONCO^®^ Freezone1^®^ equipment, with a vacuum of 0.017 mBar and a temperature of −35 °C for five days until the desired dryness was achieved. This allowed the obtaining of ~100 mg of total dry biomass per sample. Subsequently, the samples were macerated until a fine powder was obtained. To extract secondary metabolites, the dry biomass previously processed was used, and we analyzing the shoot (rosette) and the root separately. For this, the assisted extraction equipment Dionex ASE 350 (Accelerated Solvent Extractor) was used, using SST (stainless steel)-type cells, at a temperature of 60 °C for 5 min, a static time of 5 min, a washing volume of 30%, and a purge time of 90 s, using only HPLC (high-performance liquid chromatography)-grade methanol as the solvent, and performing two extraction cycles per sample. Once the above was done, the extracts were stored at −20 °C until later use. Subsequently, the previously obtained supernatants were concentrated with a rotary evaporator (Büchi, R100) to complete dryness, and later, by re-suspension, recovered in 300 µL of 50% methanol [20,21].

### 2.8. Non-Targeted Metabolomic Analysis Using Ultra-High Performance Liquid Chromatography and Accurate Mass Spectrometry

To carry out the identification of metabolites that could act as differential chemical markers between the 4 and 0.2 mM treatments, the methanolic extracts obtained previously (shoot and root) were used. Extracts (500 μL) of each extraction were taken and placed in 1.5 mL microtubes. Subsequently, 5 μL of 0.1% formic acid was added to each sample as an ionizing agent. Finally, 300 μL of each sample was taken and placed into 1 mL glass UPLC (ultra performance liquid chromatography) vials for comparative analysis of their metabolic profiles (non-targeted study). This analysis was performed using a Waters™ class I chromatographic system coupled to a Synapt G2-Si (Waters™, Milford. MA, USA) mass spectrometer of the quadrupole (Q) and time of flight (TOF) type, with an electrospray ionization source (ESI), in negative and positive mode, and in high-resolution mode, following the experimental conditions previously reported [22,23].

### 2.9. Statistical, Diversity, and Chemometric Analyses

The statistical, diversity, and chemometric analyses were carried out following the methodology proposed by Cadena-Zamudio et al. (2022) [24]. Briefly, this methodology consists of a systematic four-step process, including (i) a population analysis of metabolites’ α diversity, richness, and evenness; (ii) a chemometrics analysis to identify discriminant groups; (iii) differential metabolic marker identification (chemical marker); (iv) and enrichment analyses and annotations of active metabolic pathways enriched by differential metabolites.

#### 2.9.1. Identification of Paired Contrasts and Differential “Metabolites” Represented by Mass-to-Charge Ratio (m/z) Values Detected at a Specific Retention Time (rt)

Pairwise comparisons were made to identify differentially accumulated m/z_rt. Since the 2D PCA (2-dimensional principal component analysis) analyses [25] were not robust enough to give a clear separation between those variables that explained the majority of differences between samples, pairwise comparisons were defined based on statistical evidence obtained using a machine learning approach [26] and implementing decision tree analysis models [27,28]. It was found that nitrate concentration and genotype are the most critical variables affecting the occurrence of variance in this model, leaving aside the variables of time and organ (see related results in the corresponding section). Based on the statistical support previously described, pairwise comparisons were established as follows: first, to each ESI mode, the same time–concentration–genotype–organ was compared; that is, 15dag_4mM_WT_rosette versus (vs.) 15dag_0.2mM_WT_rosette, 30dag_4mM_WT_rosette vs. 30dag_0.2mM_WT_rosette, and so on, giving a total of four contrasts by ESI mode (Table S3).

Fold change (FC) analyses [29] were performed to obtain the differential m/z_rt. Volcano plots for each ESI mode were generated, and a value of FC ≥ 2 on each contrast was used as the threshold. Once the differential m/z_rt of each ionization mode was obtained, the resulting mass spectral libraries from both ESI modes and belonging to both sampling points (15 and 30 dag) were merged, reducing the contrasts from eight to four, ordered as follows: 15−30dag_4mM_WT_rosette vs. 15dag_0.2mM_WT_rosette, 15−30dag_4mM_WT_root vs. 15dag_0.2mM_WT-root and so on (Table S3). This was made to obtain a higher amount of differential m/z_rt and, as such, higher metabolome coverage, thus generating a more robust reconstruction of the metabolic pathways by the enrichment of chemical compounds. Notice that merging both databases from the beginning and obtaining from them the differential m/z_rt could allow us to omit relevant information provided by the contrasts of each ESI mode, a kind of bias or tendency also called mutual suppression effect [30,31]. We consider that it is possible to obtain an overview of the behavior and adaptation of the metabolome of *A. thaliana* under the proposed model because it is not limited to the realization of contrasts between ionization modes, but also allows us to see the plastic behavior of the study model in a global way (Table S3).

#### 2.9.2. Metabolic Pathway Reconstruction, Functional Enrichment Analysis, and Presumptive Annotation

The statistical analyses, metabolic pathway reconstruction, functional enrichment and presumptive annotation were carried out with the statistical software R Studio version 4.3.0 [32], using the libraries “KEGGREST, KEGGgraph, pathview, circlize, and ggplot2” and MetaboAnalyst [24,33,34].

#### 2.9.3. Tentative Identification of Differential Metabolites (Chemical Markers) between Binary Classes

To identify the behavior of several differential metabolites putatively annotated, a comparative analysis was performed by implementing a population pyramid approach. This allowed us to analyze the behavior based on the rate of change of a specific SMe in the pairwise comparisons, thus allowing us to identify its plasticity and adaptation in a general way to different abiotic stress conditions (N-starvation) over time. The software R Studio version 4.3.0 [32] and the libraries “ggplot2, dplyr and viridis” were used for these purposes.

## 3. Results

### 3.1. Establishment of Hydroponic Cultures in a Time-Course Experiment Using the WT Genotype and Loss-of-Function Double Mutant (nia1/nia2) of A. thaliana

The growth of A. thaliana seedlings from both genotypes (WT and *nia1*/*nia2*) was successful in all hydroponic chambers. The viability, germination, and seedling growth were comparable and synchronous for all sown seeds and all biological replicates used in this study (Figures S2–S4).

### 3.2. Effect of Stress Induced by Nitrate Deficiency on the Growth of the WT Genotype and Loss-of-Function Double Mutant (nia1/nia2) of A. thaliana

**Figure 1:**
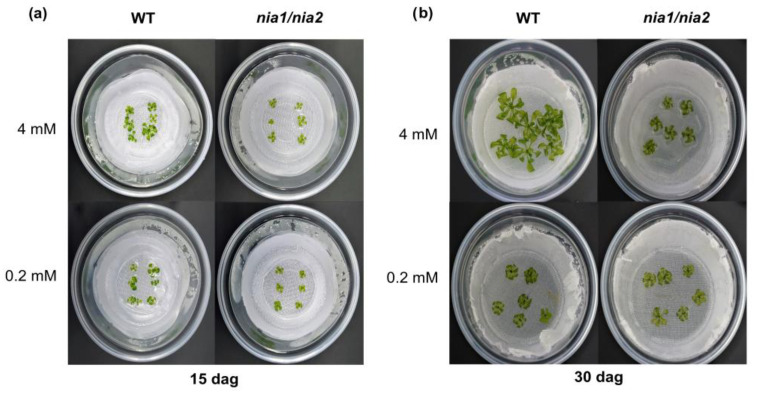
Growth contrasts between the WT genotype and the double mutant *nia1*/*nia2*. The top view of hydroponic chambers shows the growth of the WT genotype and loss-of-function double mutants *nia1*/*nia2* at (**a**) 15 dag and (**b**) 30 dag, with larger WT rosettes at both nitrate concentrations (4 and 0.2 mM). The arrested growth of *nia1*/*nia2* double mutants can be observed even at the optimal nitrate concentration (4 mM). According to the notion of proportion, the diameter of the hydroponic chambers is 12 cm.

The hydroponic cultures established at different nitrate concentrations (4 and 0.2 mM) were monitored at 15 and 30 dag to evaluate the nutritional stress induced by nitrate deficiency. We evaluated growth contrasts between the WT genotype and the *nia1*/*nia2* double mutant, and observed that WT plants achieved more development than *nia1*/*nia2* plants at both sampling times, showing significant differences according to nitrate concentrations (4 or 0.2 mM). In contrast, the loss-of-function double-mutant *nia1*/*nia2* exhibited less development than the WT genotype under all tested conditions. The difference in accumulated biomass between the two genotypes was more evident in 30-day-old seedlings. The *nia1*/*nia2* double mutant showed arrested growth independently of the availability of nitrates (Figure 1a,b).

### 3.3. Evaluation of the Effect of Nutritional Stress Induced by Nitrate Deficiency on the Growth of the Rosettes of WT and nia1/nia2 Genotypes

**Figure 2:**
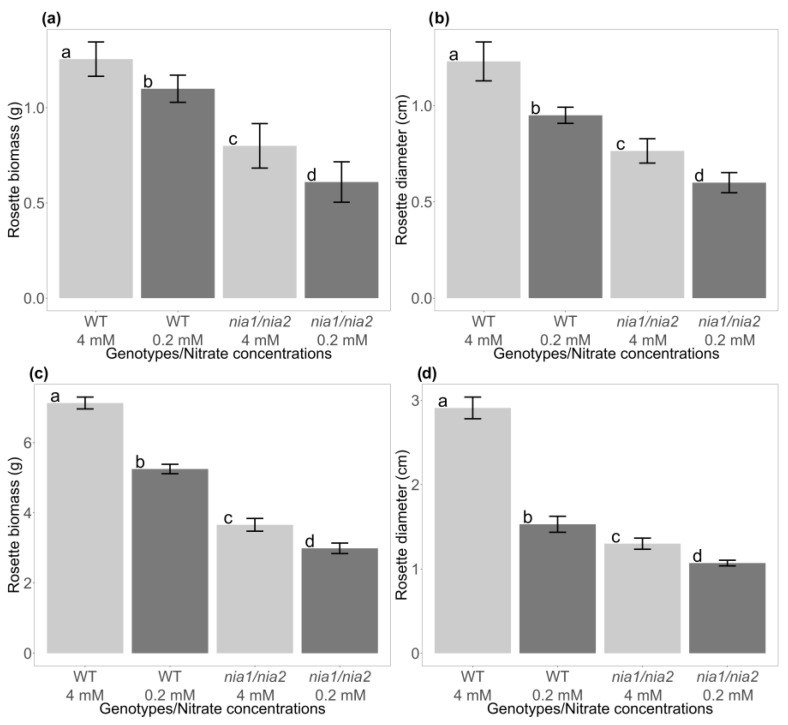
The rosette (aerial part) growth of WT and double mutant *nia1/nia2 A. thaliana* genotypes. (**a**) Fresh net biomass and (**b**) rosette diameter after 15 days of growth of seedlings in a medium supplemented with optimal and limited concentrations of N (4 and 0.2 mM, respectively) ((**c**,**d**) represent the same metrics but at 30 dag). Bars represent the average values of 18 replicates ± standard deviation. The small letters in each bar represent the one-way ANOVA results and Tukey’s *post hoc* test clustering.

The phenotypes described above were corroborated by quantifying the total fresh biomass obtained at both nitrate concentrations (4 and 0.2 mM) at each sampling time (15 and 30 dag). At 15 dag under the control condition (4 mM), the WT genotype generated more biomass (36.3%) than the *nia1*/*nia2* double mutant (Figure 2a). As for severe N stress (0.2 mM), a decrease in biomass was observed in both genotypes compared to the control condition; however, the most considerable effect was observed in the *nia1*/*nia2* genotype, which showed decreased growth compared to WT seedlings, generating 44.5% less biomass under the severe stress condition (Figure 2a). At 30 dag, the previously described behavior was sustained, as the *nia1*/*nia2* genotype at the 4 mM concentration obtained 48.7% less biomass than its WT counterpart. The same phenomenon was observed at a concentration of 0.2 mM, at which the *nia1*/*nia2* genotype obtained 43.1% less biomass than the WT genotype (Figure 2c). The results reported above were validated by statistical analysis, identifying significant differences in biomass generated between the two genotypes and under both nitrate concentrations (4 and 0.2 mM) at 15 and 30 dag (F_0.05_ = 11.81; α = 0.05; F_0.05_ = 34.34; α = 0.001). Following measurements of the shoot (rosette), pertinent statistical analyses were carried out with respect to its size (diameter), identifying a behavior similar to that described above: at 15 dag at both nitrate concentrations, the WT genotype had a greater diameter, especially under the control concentration (4 mM), followed by the severe stress condition (0.2 mM) (Figure 2b). The *nia1*/*nia2* double mutant presented a lower rate of growth at both concentrations (Figure 2b). The results reported above were validated by statistical analysis, identifying significant differences concerning the rosette diameter obtained between the genotypes and nitrate concentrations at 15 dag (F_0.05_ = 19.33; α = 0.001). At 30 dag (Figure 2d), the behavior was sustained, as the WT genotype had the largest rosette diameter at both nitrate concentrations, followed by *nia1*/*nia2* (4 and 0.2 mM, respectively). The differences were also statistically significant (F_0.05_ = 19.75; α = 0.001). We conclude that the 4 mM concentration contributed the most to the increase in biomass and diameter at both sampling points (15 and 30 dag) and for both analyzed genotypes (WT and *nia1*/*nia2*). Under the severe stress concentration (0.2 mM), the WT genotype exhibited the most outstanding development, followed by the *nia1*/*nia2* double mutant, for which the optimal concentration (4 mM) “resembles” the development exhibited by the WT genotype under the severe stress condition (0.2 mM) (Figure 2a–d).

### 3.4. Evaluation of the Effect of Nutritional Stress Induced by Nitrate Deficiency on the Growth of the Root System of WT and nia1/nia2 Genotypes

**Figure 3:**
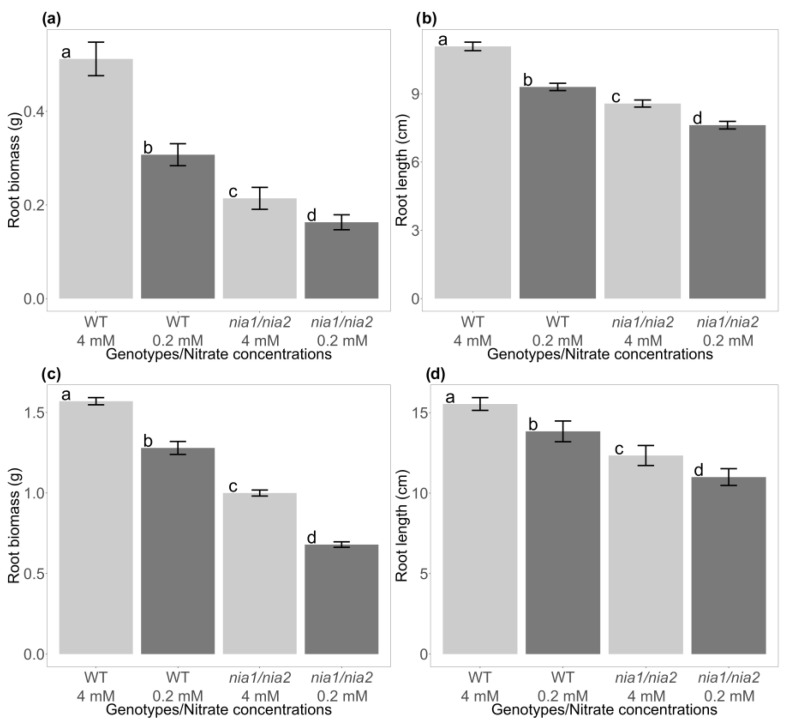
Root growth of WT and double mutant *nia1/nia2 A. thaliana* genotypes. (**a**) Fresh net biomass and (**b**) root length after 15 days of growth of seedlings in a medium supplemented with optimal and limited concentrations of N (4 and 0.2 mM, respectively) ((**c**,**d**) represent the same metrics but at 30 dag). Bars are average values of 18 replicates ± standard deviation. The small letters in each bar represent the one-way ANOVA results and Tukey’s *post hoc* test clustering.

Regarding root growth, the amount of fresh biomass obtained at each sampling time was weighed (Figure S4a,b). Results similar to those obtained from the rosettes were obtained, with the WT genotype generating the most biomass at 15 and 30 dag under both nitrate concentrations (4 and 0.2 mM) (Figure 3a,c). At 15 dag, the *nia1*/*nia2* double mutant presented 58% less biomass than the WT genotype generated at the control concentration (4 mM) and 47% less under the severe stress condition (0.2 mM) (Figure 3a). At 30 dag (and compared to the previous sampling point), both genotypes (WT and *nia1*/*nia2*) showed increased biomass. However, the differences observed between genotypes and the nitrate concentrations were preserved, with the WT genotype generating the most root biomass (Figure 3c). Statistical analyses corroborate the significance of the observed differences, supporting the results reported above (F_0.05_ = 8.85; α= 0.001; F_0.05_ = 31.32; α= 0.001) for the variables, the sampling times (15 and 30 dag), and the nitrate concentrations (4 and 0.2 mM). As described for the rosettes, quantifications and ANOVA analyses (one-way) were performed to identify statistically significant differences not only in the generated biomass, but also in the length of the primary root (Figure S4a,b). We observed that biomass statistically significantly differed at 15 and 30 dag at both nitrate concentrations (Figure 3b,d). Once again, the *nia1*/*nia2* double mutant achieved the least elongation of the root system, whereas the WT achieved the greatest elongation of the root system at both sampling points at 15 and 30 dag (Figure 3b,d) (F_0.05_ = 9.51; α = 0.001; F_0.05_ = 45.48; α = 0.001). The 4 mM concentration allowed for the greatest biomass generation and root elongation at both analyzed sampling points (Figure 3a–d). Owing to its limited capacity to reduce nitrates, the development of the *nia1*/*nia2* double mutant is affected even under optimal nitrate conditions (4 mM).

### 3.5. Metabolomic Compositional Analysis as a Function of α Diversity, Richness, and Equity of Metabolites (m/z_rt)

#### 3.5.1. Acquisition of Spectrometric Signals

The complete spectrum of *A. thaliana* was obtained in positive and negative ESI modes, which were generated using an ultra-high-resolution mass spectrometry platform. Both ESI modes (ESI^+^ and ESI^−^) were used to avoid bias and erroneous conclusions and improve metabolome coverage. Although the ESI^+^ mode has generally been favored in many previous studies, recently, ESI^−^ has been proven to be a preferrable option owing to its ionization efficiency, which increases its detection limits [35]. Furthermore, in a previous study, we demonstrated that the ESI modes are complementary and not mutually exclusive [24]. In the present study, we obtained 7928 signals (spectrometric characteristics)—3407 from ESI^+^ and 4521 from ESI^−^ (Table S4 and Table S5, respectively). Each signal represents an ion with a specific m/z_rt and ion abundance (count). All unannotated and suspected metabolites (m/z_rt) were analyzed following the steps described below.

#### 3.5.2. Suspected Metabolite (m/z_rt) Diversity Analysis by Species Accumulation Curves (SACs)

**Figure 4:**
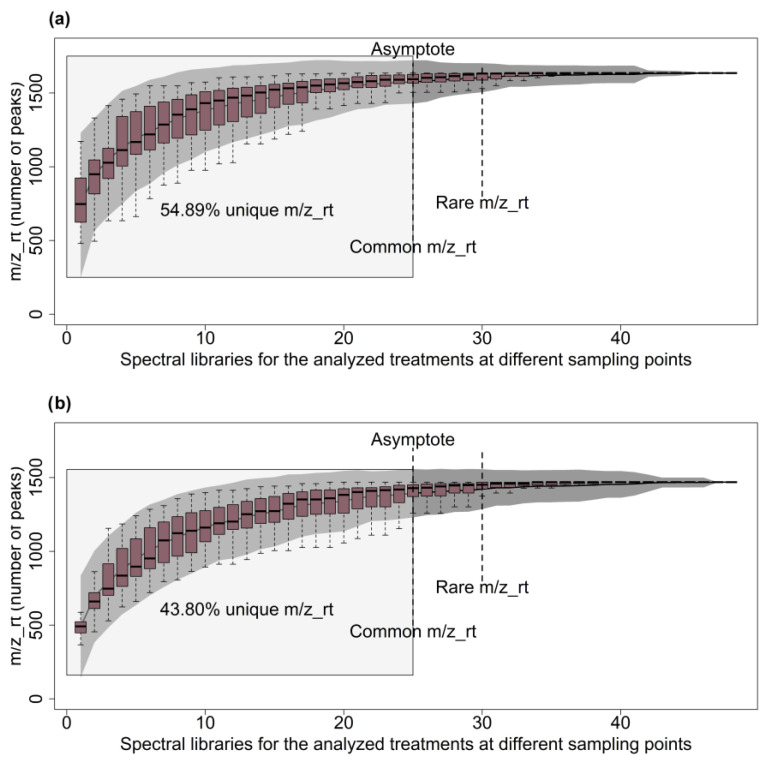
m/z_rt accumulation curves of the *A. thaliana* metabolome. (**a**,**b**) Positive and negative ESI modes (ESI^+^ and ESI^−^, respectively). The limit of common m/z_rt is defined as the point at which the shoulder of the curve (width) reaches the asymptote. Even when the curves are similar, the *A. thaliana* metabolome obtained in ESI^+^ mode is much more diverse than that obtained in ESI^−^ mode. The box plots show the median and SD for the smallest sample size. The light gray box represents the area where the unique mz/rt are found, contributing most of the diversity of the *A. thaliana* metabolome for both ESI modes.

Recently, we have proven that some approaches, concepts, terms, and ecological-type analyses can help to analyze the diversity of spectrometric features (m/z_rt) as part of a complex metabolome. Estimators of diversity, richness, and abundance of species usually applied in community and population ecology studies can be used to analyze the metabolomes by treating each spectrometric feature (m/z_rt) in the metabolome as a species belonging to a diverse community. Under this guidance and using the Chao1, Jack 1, and Jack 2 indices as parametric estimators, the m/z_rt composition of the *A. thaliana* metabolome was first analyzed using species accumulation curves (SACs) (see [24] for more details). In total, 3407 and 4521 spectrometric features (m/z_rt) were obtained in the ESI^+^ and ESI^−^ modes, respectively, representing the calculation and graphing of the average number of species (and the SD of the smallest sample size). The maximum accumulation of m/z_rt was observed under the N-deprivation condition (0.2 mM) at 30 dag in the WT genotype roots and using ESI^+^ mode. Once the accumulation curves reached the asymptote, the curve comprised 1172 species (34.39% of the total m/z_rt pairs identified from the mass spectra generated in ESI^+^ mode). The metabolomes with uncommon m/z_rt were those obtained at 30 dag from the rosettes of the *nia1*/*nia2* double mutant at both nitrate concentrations (4 and 0.2 mM), showing a total of 495 and 480 m/z_rt, respectively (Figure 4a; Table S6). The above findings demonstrate that the ESI^+^ SACs initially presented a high proportion of relatively abundant species, with an initial steep ascending slope and an early plateau, indicating that uncommon m/z_rt were the least detectable in this ionization mode.

The analysis conducted using ESI^−^ mode showed that *nia1*/*nia2* shoots growing at 4 mM were the most chemically diverse sample with the greatest number of m/z_rt (a total of 848 m/z_rt at the asymptote). The least rich metabolome or lowest diversity of m/z_rt corresponded to the WT genotype roots growing under N starvation harvested at 30 dag (366 m/z_rt; Figure 4b; Table S7). In the ESI^−^ ionization mode, the SACs’ behavior differed from that observed in ESI^+^ mode, showing a moderately high rate of common m/z_rt and a relatively high representation of rare m/z_rt. The results described above allow us to conclude that the two ESI ionization modes behave similarly to a certain extent, given the common spectrometric features accounting for a large part of the beginning of the curve and the rare spectrometric features accounting for a minimal part of the end of the curve. Such a phenomenon is an effect of time and the concentration of nitrates, as with increased exposure to the tested nitrate concentrations (i.e., 30 dag), the diversity of m/z_rt was reduced (Tables S5 and S6).

#### 3.5.3. Nitrate Starvation as a Modulator of the α-Diversity, Richness, and Evenness of m/z_rt Pairs in the Metabolome of *A. thaliana*

**Figure 5:**
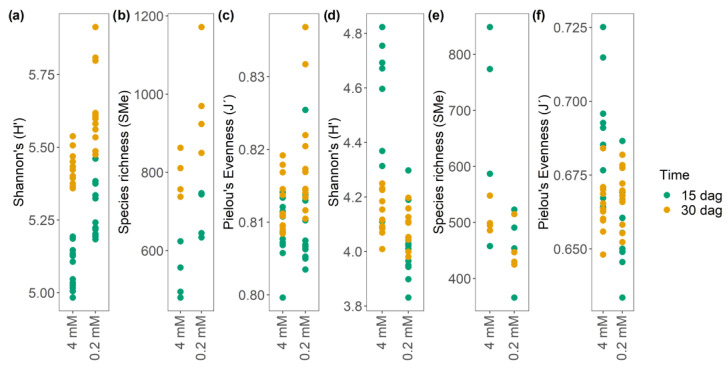
Diversity estimators of the *A. thaliana* metabolome in both ionization modes (ESI^+^ and ESI^−^). Estimates of α diversity (Shannon’s H’), species richness, and Pielou fairness (J’) for the metabolome in ESI^+^ ((**a**), (**b**), and (**c**), respectively) and ESI^−^ ((**d**), (**e**), and (**f**), respectively) modes. The metabolome in ESI^+^ (and concerning ESI^−^) mode is much more diverse, with considerable richness and equity regarding the distribution of m/z_rt at both N concentrations (2 and 0.2 mM) and time points (15 and 30 dag), indicating the clear dominance of the m/z_rt in ESI^−^ mode.

Alpha diversity (α diversity) analyses were performed using Shannon’s H’, species (m/z_rt) richness, and Pielou (J’) evenness or equity as parameters for the datasets obtained in both ESI modes in order to elucidate the dynamics of the complex metabolomes obtained in this study. Diversity indices are statistical measures used to characterize richness (the number of species) and evenness (uniform abundance). The α diversity estimator (Shannon’s H’) showed that the metabolome of *A. thaliana* obtained in ESI^+^ mode is more diverse (5 to 5.9) than that obtained in ESI^−^ mode (3.8 to 4.69) (Figure 5a,d; Tables S5 and S6), as reflected by the tested nitrate concentrations (4 and 0.2 mM) and sampling points (15 and 30 dag). For ESI^+^ mode, the N starvation treatments (0.2 mM) at 30 dag resulted in the highest H’ values (Figure 5a), in contrast with the ESI^−^ mode, for which the optimal concentration (4 mM) at 15 dag achieved the highest values of α diversity.

Regarding the N starvation condition (0.2 mM), the two sampling times (15 and 30 dag) are similar in terms of their diversity (Figure 5d). Concerning the species richness (dominance) (Figure 5b,e), the metabolome obtained in ESI^+^ mode shows a greater species richness at 30 dag at both tested nitrate concentrations (Figure 5b). However, the opposite results were obtained when analyzing ESI− mode, with the greatest species richness found at 15 dag and a concentration of 4 mM, and more homogenous results under the N starvation treatment (0.2 mM) (Figure 5e). The findings reported above are supported by the analyses carried out using Pielou’s equity index (J’), which, in ESI^+^ mode, shows a considerably more equitable community at the control concentration (4 mM) than at the limiting concentration of nitrates (0.2 mM) (Figure 5c). This finding is consistent with the two previous analyses of H’ and richness, which showed a clear dominance of certain species (m/z_rt) under the N starvation condition. In ESI^−^ mode, J’ is also consistent with H’ and richness, with N starvation achieving the most equality and homogeneity of species under the optimal N condition (Figure 5f). The results reported above enabled the elucidation of an “explicit population pattern” in the metabolome of *A. thaliana*, as the behavior observed in ESI^+^ mode is the opposite of that observed in ESI^−^ mode, which suggests plastic metabolic adaptation induced by abiotic factors such as N availability over time. Additionally, to verify whether abiotic stress (and not another variable in the system) governs the diversity of m/z_rt identified in both ionization modes, we generated a rank–abundance curve (RAC). The results show that in both ESI modes, the diversity and richness of m/z_rt fit the null model over time, confirming that the patterns observed in the metabolome are not caused by stochastic factors but by factors induced over time, at least on *A. thaliana* hydroponic cultures (Figure S5).

**Figure 6:**
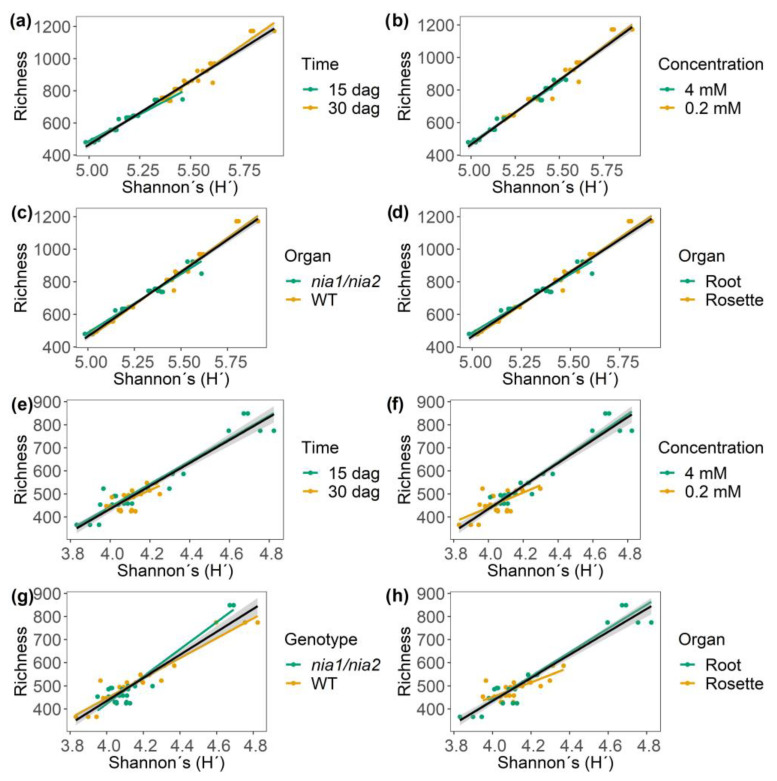
Pearson correlation and covariance between the richness and diversity of the *A. thaliana* metabolome in both ESI modes: (**a**–**d**) ESI^+^ mode; (**e**–**h**) ESI^−^ mode. Positive correlative and covariance values were obtained in both modes. The yellow and green lines correspond to the linear model of the correlation for each variable. The black line corresponds to the optimal theoretical linear distribution model, and the gray shading indicates confidence intervals (95%).

Subsequently, a one-way analysis of variance (ANOVA) was performed in order to determine whether the differences observed in the diversity estimators, specifically the H’ of the metabolome of *A. thaliana*, are statistically significant, as the main factor from which the other estimators are derived. ESI^+^ mode showed significant differences in α diversity (F_0.05_ = 66.16; α = 0.001) at the late sampling time (30 dag), contributing to such differences and confirming the assumptions mentioned above, as 30 dag achieved optimal results for all calculated diversity estimators (Figure 5a–c and Figure 6a). Significant differences were also found regarding α diversity and nitrate concentrations (also in ESI^+^ mode), with the N starvation condition (0.2 mM) leading to the greatest distinction (F_0.05_ = 66.16; α = 0.001), which is consistent with the results described above (Figure 5a–c and Figure S6b). Analysis of variance revealed statistically significant differences in terms of time in ESI^−^ mode, with the most evident distinction observed at 15 dag (F0.05 = 3.72; α = 0.05) (Figure S6c). The results achieved at a 4 mM nitrogen concentration significantly differed from those achieved under the other concentrations (F0.05 = 18.73; α = 0.001) (Figure S6d). Finally, taking the WT genotype as a reference point under the stress concentration (0.2 mM), the *nia1*/*nia2* double mutant shows significantly greater diversity both at the optimal nitrate concentration (4 mM) (F0.05 = 8.32; α = 0.05) (F0.05 = 5.03; α = 0.05) and at the limiting concentration of N (0.2 mM) (F0.05 = 6.63; α = 0.05) (F0.05 = 4.30; α = 0.05) in both ionization modes.

Independent of nitrate concentration, *nia1*/*nia2* plants cannot reduce nitrate, so their metabolic and physiological responses mimic the nitrate starvation response [36,37]. Stress in a plant does not necessarily increase/reduce the amounts of metabolites, but can alter their type, concentration, and composition. A considerable amount of evidence suggests that several SMes can be synthesized in response to stress conditions (e.g., those synthesized as part of the defensive arsenal of plants). However, based on the availability of substrates, many such metabolites may be synthesized instead of others that promote or are related to growth, with plants compromising growth in order to prioritize defensive response.

#### 3.5.4. Identification of Correlation Patterns between α Diversity and Richness Equity (J’) of m/z_rt over Time and Across Nitrate Concentrations

Pearson’s correlation (r) and covariance (R^2^) analyses were performed in order to verify the patterns described above (H’, richness, and J’) using a rearrangement based on the variable under study (time, concentration, genotype, or organ) to identify the behavior and distribution of the m/z_rt based on the variable of interest. Therefore, we were able to elucidate that in ESI^+^ mode, richness is positively correlated with a relatively high percentage of covariance, depending on the diversity (H’), with r = 0.98 and R^2^ = 96.6%, and a *p* value ≤ 0.05 (Figure 6a–d). For the time variable, the maximum diversity in terms of richness was achieved at 30 dag (Figure 6a). For the nitrate concentration variable, at a concentration of 0.2 mM, the richness and diversity are influenced by other variables (Figure 6b). With respect to the genotype variable (WT and *nia1*/*nia2*), both genotypes benefited from the conditions established in the time-course experiment. However, the richness and diversity were the greatest in the WT genotype. Finally, in relation to the organ variable (shoot vs. root), shoots corresponded with the greatest observed m/z_rt diversity.

Regarding the ESI^−^ mode (Figure 6e–h), as in ESI^+^ mode, positive correlative and covariance values were found (r = 0.94; R^2^ = 89.0 %; *p*-value ≤ 0.05). The behavior of the m/z_rt depends on the variables being analyzed. Richness and diversity were most influenced at 15 dag (Figure 6e). However, for the concentration variable (Figure 6f), the control condition (4 mM) was the most predominant in terms of richness and diversity. For the genotype variable (Figure 6g) (as in ESI^+^ mode), the two genotypes had the same influence on metabolic richness and diversity. Finally, the organ variable (Figure 6h) presented a behavior opposite to that in ESI^+^ mode, as in ESI^−^ mode, the organ that governed the metabolic richness and diversity was the root and not the shoot.

Positive correlative and covariance values were identified (r = 0.67; R^2^ = 45.1%; *p*-value ≤ 0.05) in terms of Pielou species equity (J’) (m/z_rt) in ESI^+^ mode (Figure S7a–d). The late sampling time (30 dag) was associated with the most equity in the metabolic community, as represented by an increase in diversity (Figure S7a). The stress concentrations of total nitrates available in the medium (0.2 mM) proved to positively influence equity and diversity (Figure S7b). The WT genotype exerted the greatest effect on equity (Figure S7c). Finally, the shoots were associated with the greatest equity in terms of diversity (Figure S7d). Positive correlative values were also identified for all analyzed variables (r = 0.94; R^2^ = 89.0%; *p*-value ≤ 0.05) in ESI^−^ mode (Figure S7e–h). The 15 dag time point proved to be more equitable than the later time point (30 dag; Figure S7a). The control concentration (4 mM) resulted in the greatest diversity and, therefore, the greatest equity (Figure S7b). The WT and *nia1*/*nia2* double mutant genotypes showed similar behaviors, although the WT genotype was more equitable (Figure S7g). Finally, roots were found to be more equitable in terms of the organ variable than the shoots (Figure S7h) in both ESI modes. Analyses of correlation and covariance revealed a distinct difference in the metabolic diversity of *A. thaliana*. Specifically, in ESI^+^ mode, the metabolic diversity and richness were found to be most significantly influenced by the variables at 30 dag, especially at a stress concentration of 0.2 mM in shoots of the WT genotype. The opposite was observed in ESI^−^ mode, as the early time point (15 dag), the control concentration (4 mM), and the roots of the WT genotype (same as ESI^+^) were found to most directly impact richness and equity in terms of diversity. In both ESI modes, the two analyzed genotypes exerted an equal influence.

### 3.6. Chemometric Analyses to Identify Discriminant Groups in the Metabolome of A. thaliana

#### 3.6.1. Two-Dimensional Principal Component Analysis (2D PCA) for the Identification of Statistically Relevant Binary Classes

**Figure 7:**
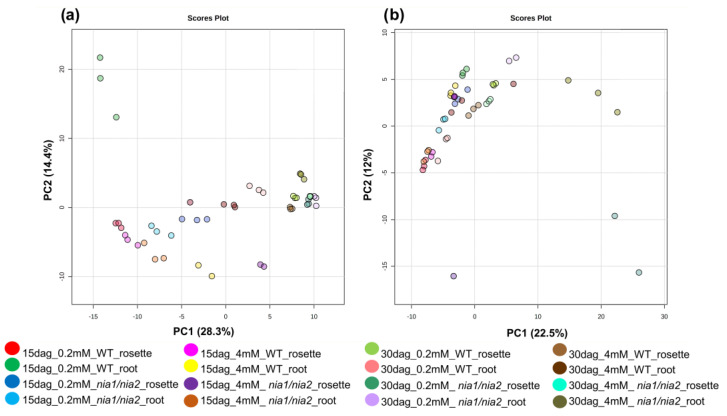
Two−dimensional PCA of the *A. thaliana* metabolome in both ESI modes. In both (**a**) ESI^+^ and (**b**) ESI^−^ modes, the percentage of variance explained by the first two principal components (PC1 and PC2) is less than 30% (around 25 and 15%, respectively).

Data obtained from spectral libraries were normalized using no-scale values and the quantile transformation and log transformation methods because they generally improve data interpretability by reducing skewness [38,39]. For both ESI modes, the range of normalized intensities obtained from each detected m/z_rt showed a normal distribution (or Gaussian distribution; Figure S8). Once data were normalized, in order to identify trends or discriminant groups responsible for the highest percentage of variance, unsupervised multivariate analyses were implemented, visualizing the multidimensional relationships of the measured variables, that is, each m/z_rt obtained under predefined conditions (N concentration (4 or 0.2 mM), sampling point (15 or 30 dag), genotype (WT or *nia1*/*nia2*), and organ (rosette or root)). Principal component analysis (PCA) did not show clearly distinguishable discriminating groups in either ESI^+^ or ESI^−^ mode, which may explain the high percentage of variance (28.3 and 14.4% for principal component one (PC1) and 22.5 and 12% for PC2, respectively) (Figure 7 and Figure S9; Tables S8 and S9). The results were corroborated by the 2D PCA biplot analysis (eigenvalue and eigenvectors), with no clear distinction observed between the m/z_rt and the sample groups in either ESI^+^ or ESI^−^ mode (Figure S10).

Considering that the percentage of variance explained by the first two or three principal components (PC) in PCA was less than 30% and no discriminating groups were distinguishable, we generated heat maps by implementing hierarchical clustering methods with a non-disruptive approach using Ward’s algorithm and Pearson correlation (Figure S11). Under the considered scenario, libraries were not clustered into discriminating groups partially or fully associated with the variables such as N concentration, sampling point, organ, or genotype. Scenarios like those described above indicate a bias toward significance among variables or non-linearity in the data, i.e., the analyzed variables (independent and/or dependent) may not necessarily be linearly related. The metabolome of *A. thaliana* may also be a considerably heterogeneous community, with the variables themselves capable of influencing the variance in a large percentage equitably; in other words, the variance was evenly divided among the four established experimental variables (time, concentration, genotype, and organ), and none of them were considered highly significant. Based on these observations and leaving aside the unsupervised models, a predictive model was implemented through the analysis of decision trees, showing that in both ionization modes, the genotypes and differences in N concentrations were the variables that contributed the most to the variance in the experimental design (Figure S12). A pairwise comparison of the spectral libraries obtained for each ESI mode was performed based on this statistical support, as previously described (see Section 2 Methods for details).

#### 3.6.2. Identification of Differential m/z_rt (Chemical Markers) by Fold-Change (FC) Analysis

In order to identify differentially synthesized/accumulated m/z_rt, pairwise comparisons of spectral libraries were performed. Comparison pairs (Table S3) were defined based on the statistical results described in the previous sections (see Section 2.9.1, Section 3.3, Section 3.4 and Section 3.5.4 and Figure S12). Once the contrasts were carried out, fold-change (FC) analyses were performed to identify differentially synthesized/accumulated m/z_rt. A fold-change value (FC) ≥ 2 was used as a threshold (Table S10; Figure S13). In ESI^+^ mode, 1818 m/z_rt were identified as differentially synthesized/accumulated, among which 998 were identified in a single comparison, whereas 820 m/z_rt were identified in more than one comparison. The most differentially synthesized/accumulated m/z_rt was observed in the roots of *nia1*/*nia2* double mutants, independently of the sampling point and N concentration. In contrast, the least synthesized/accumulated m/z_rt was observed in the rosettes of the WT genotype at the late sampling time (30 dag) at both tested nitrate concentrations (Figure S14).

In ESI^−^ mode, 3214 m/z_rt were identified as differentially synthesized/accumulated, among which 1408 were identified in a single comparison, whereas 1806 were identified in more than one comparison. The most differentially accumulated m/z_rt came from seedling-grown plants, spanning both the rosette and root organs and collected at both nitrate concentrations and from both genotypes at 30 dag. In contrast, the least differentially synthesized/accumulated m/z_rt was obtained at 15 dag from the rosette of the WT genotype (Figure S14), reinforcing the results previously obtained in the diversity analyses (Section 3.5.2, Section 3.5.3 and Section 3.5.4), as the differences obtained in terms of differentially synthesized/accumulated m/z_rt in both ionization modes are consistent with the analysis of SAC, H’, m/z_rt richness, and J’. The unique m/z_rt obtained in ESI^+^ and ESI^−^ modes represent 54.89% and 43.80% of the total diversity, respectively. A less diverse “community” tends to have a lower m/z_rt richness, resulting in less equity (J’) and a clear dominance of common m/z_rt, as is consistent with observations in ESI^−^ and ESI^+^ modes and the opposite behavior of the diversity estimators.

#### 3.6.3. Reconstruction of Metabolic Pathways, Functional Enrichment Analysis, and Presumptive Annotation of Differentially Synthesized/Accumulated m/z_rt in the Metabolomes of WT and Loss-of-Function *nia1*/*nia2* Double-Mutant Genotypes

##### WT Genotype

Given that changes in the *A. thaliana* metabolome are represented by the m/z_rt identified as differentially synthesized/accumulated (independently of ESI mode), m/z_rt were first merged, then annotated, inheriting an identity from KEGG codes (see details in the Section 2 Methods section). In total, 735 m/z_rt were annotated, 315 of which were identified as differentially synthesized/accumulated in the WT genotype and 420 of which were identified in the *nia1*/*nia2* double mutant. Using these KEGG codes, metabolic pathways were reconstructed (Figure S15a–f). Of the 79 reconstructed metabolic pathways, 16 were found because annotated m/z_rt were identified as differentially synthesized/accumulated in a unique contrast; the remaining metabolic pathways (63) were enriched by annotated m/z_rt that were identified as differentially synthesized/accumulated in two or more comparisons. The pathways with the greatest presence throughout the *A. thalina* metabolome (>50% of the contrasts) were the cutin, suberin, and wax biosynthesis pathways (with at least 6 of approximately 27 metabolites involved in the pathway); the flavone and flavonol biosynthesis pathways (8 of 51); the flavonoid biosynthesis pathway (13 of 74); the glucosinolate (19 of 77), phenylalanine, tyrosine, and tryptophan biosynthesis pathways (4 of 35); the phenylpropanoid biosynthesis pathway (9 of 58); and the terpenoid backbone biosynthesis pathway (6 of 46), all of which correspond to the secondary metabolism of *A. thaliana* (Table S11). This presumptive annotation process was performed followed by metabolic pathway enrichment analysis for all m/z_rt identified as differentially synthesized/accumulated in each comparison (contrast) of a pair of samples (Table S12). The analysis assigned various differential m/z_rt to multiple pathways due to the elaborate network of interconnected pathways representing the metabolome of a plant (i), and because plant metabolites fulfill a wide range of biological functions; as such, many metabolites are involved in several biological processes, e.g., (ii) some metabolites may be essential for plant growth and development but also participate in the response to abiotic or biotic stress conditions. In some cases, an incorrect annotation may also occur, i.e., (iii) the KEGG code may be assigned incorrectly to distinct metabolites with the same m/z-rt. Further details are provided in the Methods section and in some references related to Mummichog, the Python program used to assign KEGG codes and enriched metabolites in metabolic pathways and networks to traits (m/z_rt) obtained from high-throughput untargeted metabolomics spectral libraries [40,41,42,43]. Below, we present circular graphic representations that visualize the relationships between SMes, their relative abundance, and the biosynthesis pathways in which they are involved.

**Figure 8:**
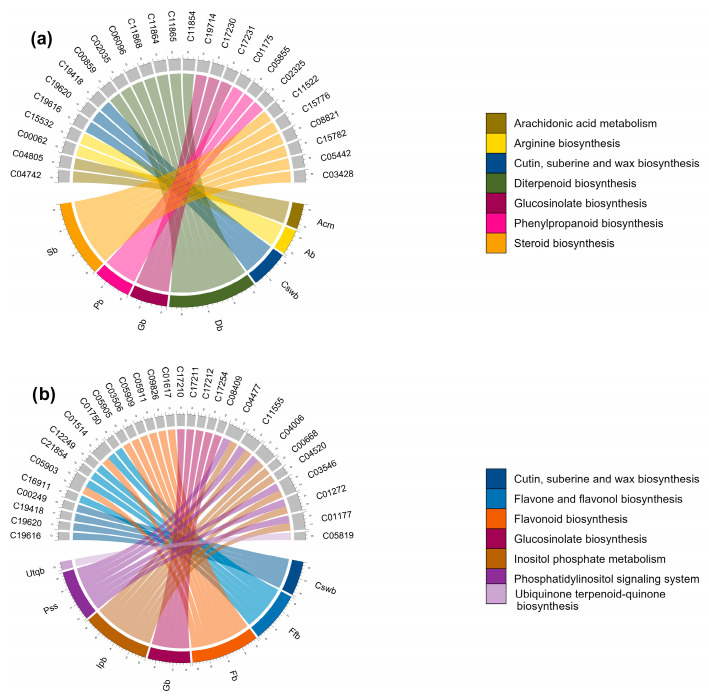

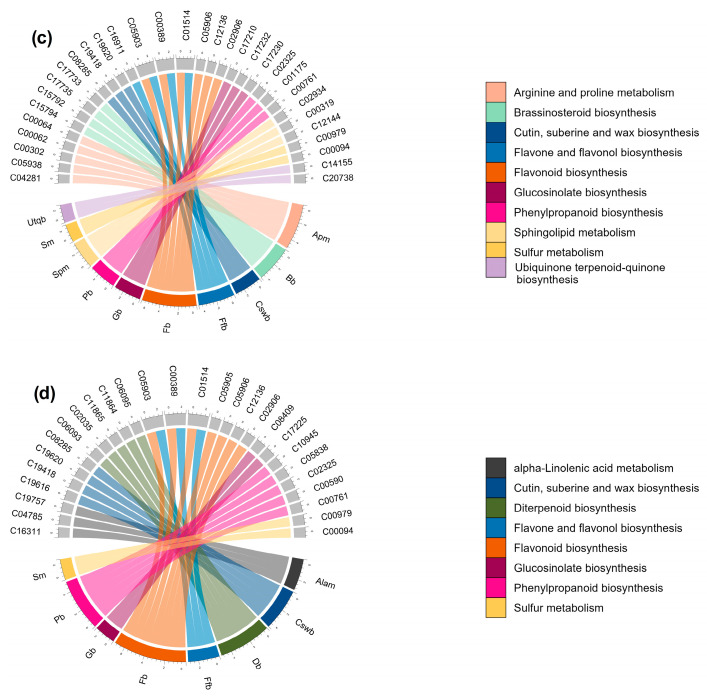
Enriched metabolic pathways in response to N availability in the *A. thaliana* WT genotype. (**a**) The metabolic pathways enriched at 4 mM in the rosettes include arachidonic acid metabolism (Acm); arginine biosynthesis (Ab); cutin, suberin, and wax biosynthesis (Cswb); diterpenoid biosynthesis (Db); glucosinolate biosynthesis (Gb); phenylpropanoid biosynthesis (Pb), and steroid biosynthesis (Sb). In contrast (**b**), the metabolic pathways enriched at 0.2 mM include cutin, suberin, and wax biosynthesis (Cswb); flavone and flavonol biosynthesis (Ffb); flavonoid biosynthesis (Fb); glucosinolate biosynthesis (Gb); inositol phosphate metabolism (Ipb); phosphatidylinositol signaling system (Pss); and ubiquinone and another terpenoid–quinone biosynthesis (Utqb). (**c**) In roots, the seven metabolic pathways most enriched when seedlings were grown at 4 mM include arginine and proline metabolism (Apm); brassinosteroid biosynthesis (Bb); cutin, suberin, and wax biosynthesis (Cswb); flavone and flavonol biosynthesis (Ffb); flavonoid biosynthesis (Fb); glucosinolate biosynthesis (Gb); phenylpropanoid biosynthesis (Pb); sphingolipid metabolism (Spm); sulfur metabolism (Sm); and ubiquinone and another terpenoid–quinone biosynthesis (Utqb). At 0.2 mM (**d**), the seven most enriched metabolic pathways include α-linolenic acid metabolism (Alam); cutin, suberin, and wax biosynthesis (Cswb); diterpenoid biosynthesis (Db); flavone and flavonol biosynthesis (Ffb); flavonoid biosynthesis (Fb); glucosinolate biosynthesis (Gb); phenylpropanoid biosynthesis (pb); and sulfur metabolism (Sm). Circos plots summarize the relationships between metabolites (annotated as m/z_rt and represented by KEGG codes) and metabolic pathways enriched by them.

Pathways corresponding to secondary metabolism and enriched by annotated m/z_rt identified as differentially synthesized/accumulated in each comparison (Table S3) are represented in the circular graphs (Figure 8). Some of them are highlighted below. For example, the shoots of WT seedlings growing at 4 mM differentially synthesized (or accumulated) 15(S)-HETE (15(S)-hydroxyeicosatetraenoic acid) and 5-HETE (5-hydroxyeicosatetraenoic), compounds from arachidonic acid metabolism that act as growth and development regulators in plants, participating in hormonal signaling, including the regulation of root growth, cell elongation, and cell division [44]. In steroid biosynthesis, compounds such as stigmasterol, avenasterol, and Δ-7-avenasterol stood out. Their primary role is as essential regulators of growth and development in plants, contributing to the structural integrity of membranes and regulating their permeability, in addition to being involved in cell elongation and the development of plant organs (including roots). They also interact with plant hormones, such as auxins, gibberellins, and brassinosteroids, which are perceived extracellularly by membrane receptors. In diterpenoid biosynthesis, primary-type terpenoids such as gibberellin A20, gibberellin A51, gibberellin A4, gibberellin A1, gibberellin A34, gibberellin A29, and gibberellin A51–catabolite were identified, most of which have functions related to growth processes and physiological regulation [45,46].

The flavone, flavonol, and flavonoid biosynthesis pathways were found to be enriched mainly in the WT genotype shoots when seedlings were grown at 0.2 mM N concentrations (Figure 8a,b). The differentially synthesized/accumulated metabolites that contribute to this enrichment include compounds with a polyphenolic structure, such as kaempferol, kaempferol 3-O-rhamnoside-7-O-glucoside, kaempferol-3-O-glucoside, and quercitrin, which have been reported to be involved in processes of protection against abiotic stress such as UV-B radiation, drought, and nutritional stress, in addition to participating in detoxification processes in reactive oxygen species (ROS) caused by such stress [47,48,49]. Pathways of the inositol phosphate metabolism and phosphatidylinositol signaling system were also enriched by compounds such as D-myo-inositol 4-phosphate, 1D-myo-inositol 3-phosphate, myo-inositol 1-phosphate, 1D-myo-inositol 1,4,5,6-tetrakisphosphate, inositol 1,3,4,5-tetraphosphate, and 1D-myo-inositol 1,3,4,6-tetrakisphosphate, among others. These SMes stand out for their essential role in tolerance to abiotic stress and are involved in the transduction processes of various signals through the addition or removal of lipids or phosphates and their derivatives, as well as in the management of ROS [50,51].

Similarly to those found in shoots, the pathways enriched by metabolites differentially synthesized/accumulated in the WT seedlings’ roots (Figure 8c,d) include those related to the biosynthesis of some compounds involved in growth and development, such as, among others, brassinosteroid biosynthesis (in which castasterone, 7-oxatyphasterol, 7-oxateasterone, and 3-dehydroteasterone were identified as differentially synthesized/accumulated metabolites) and flavonoid biosynthesis enriched by (-)-epigallocatechin, dihydromyricetin, kaempferol, and quercetin. These SMes are associated with division processes, the control of root elongation and density, and agents that modify root architecture, affecting the branching of lateral roots and the formation of root hairs, and stimulating root initiation and lateral growth [52,53,54,55,56]. Another enriched pathway at the control concentration (4 mM) was arginine and proline metabolism, with L-arginine as one of its most prominent differential metabolites, which attracted our attention because this metabolism is used as a precursor for the synthesis of nitric oxide (NO) in plants, which acts as a signaling molecule for various physiological processes, including root growth [57,58].

Regarding the metabolic pathways enriched in the roots of seedlings growing under the N-deprivation condition (0.2 mM), two routes were identified that attracted our attention. The first was the α-linolenic acid metabolism (ALAM) pathway, which is enriched by metabolites such as 13(S)-HPOT ((13S)-hydroxyoctadecadienoic acid) and (3Z)-Hex-3-en-1-yl acetate, which are essential components of membrane lipids in plants that, under stress conditions, undergo changes in their composition to maintain their integrity and function. In addition, ALAM and its derivatives, such as jasmonic acid (JA), act as signal molecules in the stress response through oxidative stress, interacting with other plant hormones, such as gibberellins, to regulate the stress response [59,60], consistent with the second pathway, diterpenoid biosynthesis, in which gibberellin A20, gibberellin A51, gibberellin A4, and gibberellin A44 diacid were found to be enriched under N starvation. These gibberellin-type metabolites are capable of modulating root growth and counteract the inhibitory effects of stress, promoting their growth under adverse conditions, as well as mitigating the oxidative stress produced by ROS and inducing the synthesis of enzymes such as superoxide dismutase (SOD) and catalase (CAT) [61,62]. The cutin, suberin, and wax biosynthesis pathways, as well as flavone and the flavonol biosynthesis, flavonoid biosynthesis, glucosinolate biosynthesis, phenylpropanoid biosynthesis, and sulfur metabolism pathways, were enriched by metabolites differentially synthesized/accumulated under either optimal or N-deprivation conditions. Many of them regulate the oxidative stress induced by ROS and are pathways whose SMes are involved in stress tolerance [55,56].

**Figure 9:**
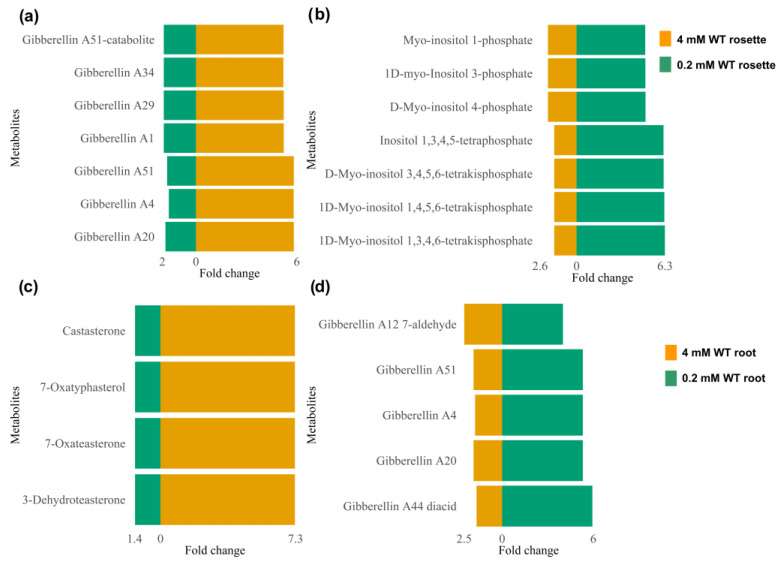
Comparative analysis of differentially synthesized/accumulated SMes mainly enriched in response to the N availability of the WT genotype (4 and 0.2 mM). Metabolites enriching the diterpenoid or brassinosteroids biosynthesis pathways with significant differences in their synthesis/accumulation in rosettes (shoots) at 4 mM and 0.2 mM (**a**,**b**) and in roots at the same concentrations ((**c**) and (**d**), respectively).

The analyses carried out in this study allowed us not only to identify metabolic pathways that are enriched by the presence of the metabolites that comprise them and that can be identified in a mass spectral library depending on the conditions that they represent (e.g., optimal or N-deprivation conditions; 4 and 0.2 mM, respectively); we also identified those metabolites that enrich pathways and show differences in their synthesis or accumulation depending on the conditions under comparison. For example, the diterpenoid biosynthesis pathway was enriched by gibberellin-type metabolites identified in shoots and roots from seedlings grown at an optimal N concentration (4 mM) (Figure 8a,c). Many of these metabolites were also found in seedlings grown under N-deprivation conditions (0.2 mM). Some of these shared metabolites showed a considerable increment in their synthesis/accumulation in the shoots of seedlings grown at an optimal N concentration (4 mM). The FC values indicate that these metabolites increased by up to six times under control conditions compared to N-deprivation (0.2 mM) (Figure 9a). Metabolites in this pathway were also increased in the roots under the N-deprivation condition (0.2 mM) (Figure 9d). A similar pattern was observed for metabolites related to brassinosteroid biosynthesis; some metabolites from this pathway showed a significant increase (FC values > 7) in roots grown at 4 mM, but were increased in shoots at 0.2 mM (FC values > 6) (Figure 9b,c). Taken together, these results indicate that in response to N-deprivation, some metabolites can be organ-differentially synthesized (or accumulated); however, once the optimal levels of N are recovered, the accumulation of these metabolites may be redistributed.

Considering the enriched metabolic pathways, as well as the differential synthesis/accumulation of metabolites, we conclude that the results obtained from mass spectral libraries are consistent with the phenotypes observed in the time course experiments, i.e., improved and sustained growth under optimal conditions but not under the N-deprivation condition (Figure 2). This result suggests that under nutritional stress, the *A. thaliana* metabolome prioritizes survival instead of growth. Furthermore, opposite patterns in the accumulation of some SMes either in roots or shoots (rosettes) may be related to the N uptake, trafficking, and homeostasis processes, which are tightly controlled in plants [63].

##### Loss-of-Function *nia1*/*nia2* Double Mutant

**Figure 10:**
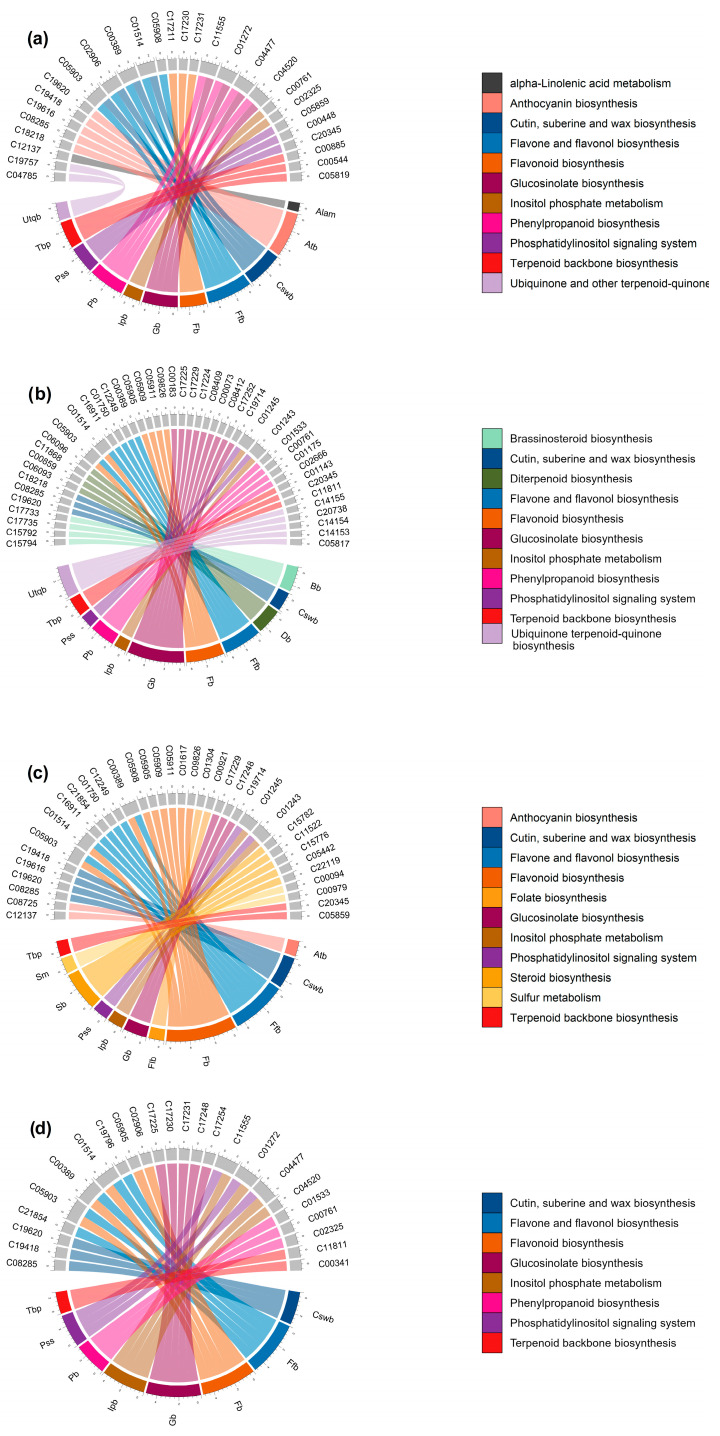
Enriched metabolic pathways in response to N availability in the loss-of-function *nia1*/*nia2* double mutant. (**a**) The metabolic pathways enriched at 4 mM in the rosettes include α-linolenic acid metabolism (Alam); anthocyanin biosynthesis (Atb); cutin, suberin, and wax biosynthesis (Cswb); flavone and flavonol biosynthesis (Ffb); flavonoid biosynthesis (Fb); glucosinolate biosynthesis (Gb); inositol phosphate metabolism (Ipb); phenylpropanoid biosynthesis (Pb); phosphatidylinositol signaling system (Pss); terpenoid backbone biosynthesis (Tbp), and ubiquinone and other terpenoid-quinone biosynthesis (Utqb). In contrast (**b**) the metabolic pathways enriched at 0.2 mM included brassinosteroid biosynthesis (Bb); cutin, suberin, and wax biosynthesis (Cswb); diterpenoid biosynthesis (Db); flavone and flavonol biosynthesis (Ffb); flavonoid biosynthesis (Fb); glucosinolate biosynthesis (Gb); inositol phosphate metabolism (Ipb); phenylpropanoid biosynthesis (Pb); phosphatidylinositol signaling system (Pss); terpenoid backbone biosynthesis (Tbp); and ubiquinone and other terpenoid–quinone biosynthesis (Utqb). (**c**) In roots, the seven metabolic pathways most enriched when seedlings were grown at 4 mM include anthocyanin biosynthesis (Atb); cutin, suberin, and wax biosynthesis (Cswb); flavone and flavonol biosynthesis (Ffb); flavonoid biosynthesis (Fb); folate biosynthesis (Flb); glucosinolate biosynthesis (Gb); inositol phosphate metabolism (Ipb); phosphatidylinositol signaling system (Pss); steroid biosynthesis (Sb); sulfur metabolism (Sm); and terpenoid backbone biosynthesis (Tbp). At 0.2 mM (**d**), the eleven most enriched metabolic pathways are cutin, suberin, and wax biosynthesis (Cswb); flavone and flavonol biosynthesis (Ffb); flavonoid biosynthesis (Fb); glucosinolate biosynthesis (Gb); inositol phosphate metabolism (Ipb); phenylpropanoid biosynthesis (Pb); phosphatidylinositol signaling system (Pss); and terpenoid backbone biosynthesis (Tbp). Circos plots summarize the relationships between metabolites (annotated as m/z_rt and represented by KEGG codes) and metabolic pathways enriched by them.

Eleven metabolic pathways were enriched in response to N availability in the rosettes from the *nia1*/*nia2* double mutant. Nine were enriched by metabolites identified under optimal and N-deprivation conditions (4 and 0.2 mM, respectively) (Figure 10a,b), including cutin, suberin, and wax biosynthesis; flavone and flavonol biosynthesis; flavonoid biosynthesis; glucosinolate biosynthesis; inositol phosphate metabolism; phenylpropanoid biosynthesis; phosphatidylinositol signaling system; terpenoid backbone biosynthesis; and ubiquinone and other terpenoid–quinone biosynthesis. SMes belonging to these pathways include farnesyl pyrophosphate, quercetin, 9,10-epoxystearic acid, kaempferol, isopentenyl phosphate, and luteolin, among many others, which stand out from the rest because they are antioxidant metabolites (and metabolic pathways) that regulate ROS-induced oxidative stress [55,56], consistent with the notion that nutrient-starved plants elicit ROS production via plant hormones such as ethylene and its signaling cascade [64].

Pathways such as α-linolenic acid metabolism and anthocyanin biosynthesis enriched by 13(S)-HPOT, cis-3-Hexenyl acetate, and pelargonidin 3-O-glucoside seem to be present mainly in the rosettes of plants growing at an optimal N concentration (4 mM) (Figure 10a). 13(S)-HPOT is a precursor of jasmonate-series lipid-derived plant hormones that trigger defense responses, including the production of secondary metabolites such as phytoalexins, phenolic compounds, and cis-3-Hexenyl acetate, which is a green leaf volatile (GVL) that plays a communication role with other plants such that its neighbors promote the synthesis of terpenes wrapped in an oxidative defense [65,66]. Pelargonidin 3-O-glucoside is another compound with potent antioxidant capacity [67]. Under the N-deprivation condition (Figure 10b), metabolites such as 3-dehydroteasterone, 7-oxatyphasterol, and 7-oxateasterone enriched the brassinosteroid biosynthesis pathway. These SMes are known to be involved in numerous responses through their interaction with other phytohormones, generating activation signals and producing antioxidants and transcription factors that respond to stress [68,69].

In contrast, the diterpenoid biosynthesis pathway with metabolites such as gibberellin A1, gibberellin A34, and gibberellin A29 was also enriched in rosettes under the N-deprivation condition (Figure 10b). These gibberellins probably act as protective molecules against oxidative stress induced in responses to N-deprivation and as mediators of plant growth in order to optimize available resources and cope with stress. This result is consistent with previous studies in which gibberellins have been proven in the responses to oxidative stress induced by salt stress (another abiotic stress condition) [70,71].

Regarding the metabolic pathways enriched in *nia1*/*nia2* roots, we found that at 4 mM, anthocyanin biosynthesis is enriched by pelargonidin 3-O-glucoside and pelargonin; steroid biosynthesis is enriched by Δ7-avenasterol, 24-methylenelophenol, 4α-methyl fecosterol, and stigmasterol; the sulfur metabolism is enriched by sulfite; and folate biosynthesis is enriched by 7,8-dihydropteroic acid, dihydropteroate, and 2,5-diamino-6-(5’-phosphoribosyl amino)-4-pyrimidineone (Figure 10c). In contrast, at 0.2 mM, the phenylpropanoid biosynthesis pathway was enriched by syringin, coniferin, and sinapyl alcohol (Figure 10d). These metabolites may help to mitigate oxidative stress [47,49]. The remaining metabolic pathways were enriched by metabolites identified under optimal and N-deprivation conditions (4 and 0.2 mM, respectively) (Figure 10c,d). Anthocyanin biosynthesis pathways attracted our attention because these SMes play an antioxidant role and repair the damage caused by oxidative stress [72]. Steroids (another enriched metabolic pathway) are also known to play roles in the regulation of root elongation and growth, as well as in the rigidity and elasticity of roots, which directly affect their capacity for growth and expansion. Steroids also help to adapt to and protect roots from abiotic stress by maintaining membrane integrity and regulating ionic homeostasis in root cells [73,74]. Finally, folates (also known as B9 vitamins) are crucial intermediates for a set of reactions that involve the transfer of single-carbon units (C1 metabolism). They are directly involved in the synthesis of nucleic acids, methionine, pantothenate, glycine, and serine, as well as, indirectly through S-adenosyl methionine, in all methylation reactions. Electron donor–acceptor interactions of folates also contribute to the production of NADPH, which is used to detoxify ROS [75,76].

**Figure 11:**
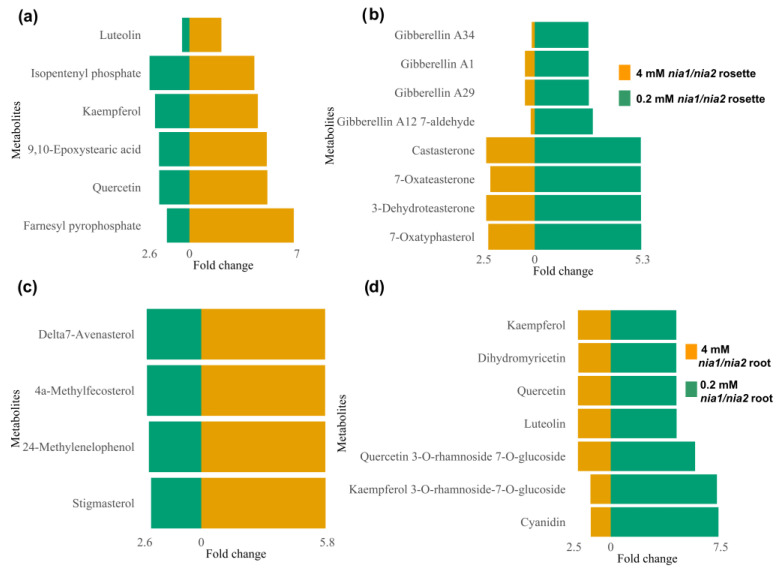
Comparative analysis of differentially synthesized/accumulated metabolites primarily enriched in the *nia1*/*nia2* double mutant in response to N availability (4 and 0.2 mM). Metabolites synthesized or accumulated with significant differences in rosettes (shoots) (**a**,**b**) and roots (**c**,**d**) at 4 mM and 0.2 mM.

Regarding the FC values and differences observed in the relative abundance of the metabolites synthesized/accumulated differentially in the analyzed organs, we noticed that farnesyl pyrophosphate, quercetin, 9,10-epoxystearic acid, kaempferol, isopentenyl phosphate, and luteolin showed increased synthesis (or accumulation) in the rosettes, which, on average, was 4.4 times higher at the optimal concentration (4 mM) than under the N-deprivation condition (0.2 mM) (Figure 11a). Figure 11b shows some metabolites also identified in rosettes but primarily synthesized or accumulated under the N-starvation condition (FC > 2.8; Figure 11b). The FC values from the metabolites identified in the roots and with considerable differences at either 4 mM or 0.2 mM are also shown in Figure 11c,d, respectively. The differences in increases ranged from 3.2 to 5.

All of the metabolites identified in both the rosettes and roots may counteract the stress caused by N-deprivation, which was expected, as the *nia1*/*nia2* double mutant is blind to N perception/availability; therefore, its development is compromised from the beginning, even when seedlings are grown under the optimal N concentration (4 mM). The phenotype of seedlings developed under optimal nitrate conditions is similar to that of seedlings grown under N-deprivation conditions (0.2 mM), as shown in Figure 2. Based on its metabolome, we suggest that the *nia1*/*nia2* double mutant continuously attempts to grow during its struggle to contain abiotic stress and achieve survival. This loss-of-function double mutant is incapable of N uptake, despite its availability, so even when growth is arrested, changes in its metabolome occur to counteract stress (as much as possible) and avoid the need to induce cell death.

## 4. Discussion

In this work, we used a hydroponic system to grow *A. thaliana* seedlings under optimal (4 mM) and N-deprivation (0.2 mM) conditions. Some phenotypic traits were analyzed in the WT genotype and a loss-of-function *nia1*/*nia2* double mutant. In the WT genotype, the rosettes and roots showed significantly less growth under the N-deprivation condition, which was evident at both early and late time points (15 and 30 dag, respectively). In contrast, the *nia1*/*nia2* double mutant showed arrested growth, even at optimal N concentrations. The growth of seedlings was “comparable” at both concentrations, although at 4 mM, seedling growth was slightly higher than that at 0.2 mM (Figure 1, Figure 2 and Figure 3, Figure S3 and S4).

The *A thaliana nia1/nia2* double mutant cannot convert nitrate into nitrite due to the loss of function of the nitrate reductase; therefore, it always shows a phenotype like that observed in N-deprivation treatments, independently of N concentration. N sensing and uptake should not be affected in the *nia1*/*nia2* double mutant; however, because the nitrate cannot be reduced, this macronutrient (N) cannot be properly assimilated. The WT genotype maintains its intact capacity to census; uses extracellular nitrogen efficiently through the activation of its signaling pathway, regulating the transcription of genes related to nitrate absorption, as well as the activity of the enzymes necessary for these functions; and is capable of adapting to variations in nitrogen availability and optimizing its growth and development accordingly [77,78].

Despite its inability to reduce N efficiently, the *nia1*/*nia2* double mutant grew at a significantly reduced rate, clearly illustrating how plants can adapt to survive, even when the availability of an essential macronutrient, such as N, is considerably reduced, because plants have developed alternative strategies to acquire nitrogen in the absence of a mechanism that allows its census, uptake, or assimilation [17,79,80]. One such strategy is the direct absorption of ammonium, which, despite its potential toxicity at high concentrations, can be directly absorbed by plants and converted to amino acids and other nitrogenous compounds without the need for enzyme nitrate reductase [81]. A lack of nitrate in plants can be compensated for by the overexpression of some high- and low-affinity ammonium transporters (AMT1- or AMT2-type transporters, respectively) [82,83]. Some of these genes are expressed mainly in roots and are induced by N starvation [84,85,86]. Depending on the species, AMT genes have been shown to have a variety of expression patterns and to be upregulated under nitrogeN-deprivation or after ammonium replenishment [87,88,89]. Another strategy is the internal reallocation of nitrogen, whereby plants can mobilize nitrogen from different organs or older leaves to younger leaves and meristems, ensuring that growing tissues have a sufficient nitrogen supply [90,91]. The preceding demonstrates how phenotypic plasticity through distinct morphological and physiological traits counteracts stress, favors survival, compromises growth, and enhances adaptive capacities.

As in previous studies, concepts and tools commonly applied in ecological studies were used in the present study to analyze the generated mass spectral libraries. Estimators of diversity, richness or abundance, and equity made it possible to conclude that mass spectral libraries obtained from both ESI modes (positive and negative) are similar but not equal, and that, as such, they are complementary. Interestingly, the metabolomes obtained in ESI^+^ (and concerning ESI^−^) mode are much more diverse and rich, but they are also more equitable in terms of m/z_rt distribution, as observed under optimal and N-deprivation conditions (Figure 5a–f). This result reinforces the idea that positive and negative ESI modes are complementary, and both are required to avoid bias and incorrect conclusions, as well as to ensure comprehension (holistic conception), mainly when metabolomes under distinct conditions (e.g., N availability, time, and genotype) are compared. Although prior to the presumptive annotation of the m/z_rt, the metabolome cannot be concluded to be more diverse when seedlings are grown under N-deprivation conditions, and based on the performed diversity analyses, we can conclude that independently of ESI mode, slightly more m/z_rt can be detected under the N-deprivation condition than under the control condition, suggesting that under N-deprivation conditions, the *A. thaliana* metabolome is more diverse in rosettes and roots, with rare increases in m/z_rt. The same occurs if the *nia1*/*nia2* double mutant (under either optimal or N-deprivation conditions) is compared with the WT grown under optimal N conditions, suggesting that when seedlings face stress conditions such as N-deprivation, their growth is arrested, with an increase in the diversity of m/z_rt (metabolites), prioritizing the synthesis (and the accumulation/abundance) of those that can be used to cope with stress, which is consistent with previous studies in which in plants, an increase in metabolomic complexity (prioritizing the availability of resources) was reported in order to cope with stress conditions and guarantee survival [14,92].

Changes in the metabolome suggest an adaptive response, i.e., the metabolism of the seedling adjusts rapidly to the N availability. This ability of plants to adjust their metabolic pathways, prioritizing the synthesis of some SMes that help to cope with stress, demonstrates their resilience and biological adaptability. In contrast to genetic alterations that occur over several generations, phenotypic (and metabolic) plasticity accounts for plant responses to environmental changes [6,7]. The resilience or inherent adaptability of the WT genotype allows it to maintain a rich and diverse metabolic profile under stress conditions [93,94]. The richness and diversity can vary between organs (e.g., rosettes and roots), consistent with previous studies in which the accumulation of intermediaries from some metabolic pathways was observed, even in an organ-specific manner [95]. The same can occur when genotypes [95,96], or developmental stages [97], are compared.

The results were obtained once mass spectral libraries were compared and differentially synthesized/accumulated metabolites (annotated m/z_rt) were identified in both the rosettes and roots. The enriched metabolic pathways suggest that the metabolomic responses that take place in response to nitrate availability are dynamic, adaptative, and complex. In the rosettes of the WT genotype, when seedlings were grown at optimal N concentrations (4 mM), metabolic pathways such as arachidonic acid metabolism, steroid biosynthesis, and diterpenoid biosynthesis were identified as enriched (Figure 8a). These metabolic pathways may be fundamentally related to growth and development, as arachidonic acid (AA), as a signaling molecule, has been proven to promote plant defense and stress responses, even eliciting cell death [98,99]. Treatments with endogenous AA promote a considerable and immediate shift in terpenoid metabolism, redirecting the pathway from higher terpenoids (i.e., steroid glycoalkaloids) to sesquiterpenoid phytoalexins [100,101]. Whereas terpenoid phytoalexins function as defense compounds against a broad spectrum of pests and pathogens, the quick shift in terpenoid metabolism can explain, at least in part, how in the absence of biotic or abiotic stress, plants can prioritize growth and development over stress response (or defense mechanisms) [102,103]. In addition to terpenoid biosynthesis, AA can also mediate some salicylic acid (SA)- and JA-dependent gene expressions [98,99,104].

In the rosettes of the seedlings (WT genotype) grown under the N-deprivation condition (0.2 mM), enriched metabolic pathways also reveal a switch, but prioritizing the nutritional stress response over growth. Pathways such as flavonoid biosynthesis, the phosphatidylinositol signaling system, and inositol phosphate metabolism were enriched mainly by metabolites identified as differentially synthesized/accumulated (Figure 8b). These pathways (and their metabolites) underline the adaptative responses of the plant to overcome abiotic stress [47,49,50]. For example, flavonoids such as quercitrin and kaempferol function as a shield against UV-B radiation, drought, and nutritional stress, and also play a role in detoxification processes in response to ROS [47,48]. Similarly, SMes belonging to the phosphatidylinositol signaling system and inositol phosphate metabolism pathways are essential for abiotic stress tolerance because they regulate antagonistic crosstalk between SA and JA. In turn, the crosstalk between SA and auxin during pathogen infection adjusts the tradeoff between immune response and plant growth [105,106]. In addition, various forms of inositol are known to effectively control the levels of ROS, which, under stress conditions, function as an alarm signal that triggers stress/defense responses through specific signal transduction pathways that involve H_2_O_2_ as a secondary messenger [107]. Antioxidant enzymes such as superoxide dismutase (SOD) and catalase (CAT) constitute the primary protection against oxidative damage and contribute to a tight control between ROS production and scavenging. This first line of defense against the effects of toxicants of high levels of ROS (in this case, the oxidative stress consequence of prolongated N-deprivation) is essential to protecting the photosynthetic machinery [50,108], which is consistent with the observed differences in the FC values of some differentially synthesized/accumulated metabolites. For example, the considerable increase in the biosynthesis of gibberellins-type diterpenoids (i.e., gibberellin A20, gibberellin A51, gibberellin A4, and gibberellin A44 diacid), which occurs mainly in the rosettes of seedlings grown under optimal N conditions, is opposite to the significant increase observed in the inositol phosphate metabolism that occurs in the rosettes of the seedlings grown under the N-deprivation condition. Gibberellins are plant hormones that control major aspects of plant growth, such as germination, elongation, flower development, and flowering time, and also play roles in cell proliferation, hypocotyl xylem expansion, phosphate starvation response, pathogen responses, oxidative stress response, and responses to abiotic environmental cues (reviewed in [109]). The inositol phosphate metabolism coordinates cellular responses to nutrient uptake and utilization, from growth factor signaling to energy homeostasis [110].

As in rosettes, in plant roots, the biosynthesis (accumulation) of gibberellins (gibberellin A20, gibberellin A51, gibberellin A4, and diacid gibberellin) increases, but in this case, the increase does not occur in roots of seedlings grown under optimal conditions, but in seedlings grown under the N starvation condition (Figure 8d). These data suggest that in roots, instead of promoting growth, gibberellins may counteract the oxidative stress produced by ROS, inducing the synthesis of oxidative enzymes such as SOD and CAT to regulate oxidative homeostasis [59,60,61,62]. In addition to diterpenoid biosynthesis, the α-linolenic acid metabolism (ALAM) is another pathway enriched in the roots of seedlings grown under N-deprivation conditions. One more time, we highlight the role of this metabolic pathway in the stress response, whereby some of its derivatives (e.g., JA) act as signal molecules, interacting with other plant hormones (such as gibberellins) to regulate the response to oxidative stress [60].

The roots of the seedlings grown under the optimal N condition (4 mM) were enriched (among others) by SMes from the brassinosteroids biosynthesis pathway (Figure 8c). In plants, brassinosteroids are not only involved in root cell elongation, but also in many other aspects of root development, such as the maintenance of meristem size, root hair formation, lateral root initiation, and gravitropic response, and in legumes, they are also involved in mycorrhizal nodule formation [52,53,54,55,56,111]. The enrichment in the brassinosteroids biosynthesis pathway was accompanied by increased L-arginine as one of the most prominent differentially synthesized/accumulated SMes. L-arginine is a precursor for the synthesis of nitric oxide (NO), which, in plants, acts as a signaling molecule, regulating various physiological processes, including root growth [57,58].

Regarding metabolic changes that occur in response to N availability in the *nia1*/*nia2* double mutant, eleven metabolic pathways were enriched in the rosettes of the seedlings, among which nine metabolic pathways were enriched by metabolites identified at both concentrations (4 and 0.2 mM; Figure 10a,b). The consistency of most enriched pathways was expected, as the *nia1*/*nia2* double mutant is affected by its ability to reduce available nitrates; as such, seedlings show a phenotype “comparable” to that of the WT seedlings grown under the N-deprivation condition. These metabolic pathways include cutin, suberin, and wax biosynthesis; flavone and flavanol biosynthesis; flavonoid biosynthesis; glucosinolate biosynthesis; inositol phosphate metabolism; phenylpropanoid biosynthesis; the phosphatidylinositol signaling system; terpenoid backbone pathway biosynthesis; and ubiquinone and other terpenoid–quinone biosynthesis, all of which are involved in neutralizing the damage caused by free radicals, such as superoxide (O_2_^−^), hydrogen peroxide (H_2_O_2_), hydroxyl •OH radicals, and singlet oxygen (^1^O_2_) [112,113], as demonstrated by differentially synthesized/accumulated antioxidant secondary metabolites such as farnesyl pyrophosphate, quercetin, 9,10-epoxystearic acid, kaempferol, isopentenyl phosphate, and luteolin, among others [114,115,116,117,118]. In *A. thaliana*, the suppression of the farnesyl diphosphate synthase has been reported to alter chloroplast development and trigger the sterol-dependent induction of JA-related responses. In contrast, the overexpression of this enzyme induces a cell death/senescence-like response and reduced cytokinin levels [119,120]. These results suggest that plants quickly counter changes that modify the redox balance, including those that occur under stress conditions (e.g., nutritional stress) or those related to an oxidative burst, i.e., rapid reactive oxygen species (ROS) production, which is important and required for defense signaling in all kinds of responses to pathogen attacks, and can trigger a hypersensitive response and programmed cell death [121].

The metabolic pathways enriched under the optimal concentration of nitrates (4 mM) were the metabolism of α-linolenic acid and the biosynthesis of anthocyanins (Figure 10a). In contrast, under the N-deprivation condition (0.2 mM), the enriched pathways included brassinosteroid biosynthesis and diterpenoid biosynthesis (Figure 10b). α-Linolenic acid is a major source of jasmonate hormones, which trigger defense responses, including the production of phytoalexins and phenolic compounds [122], which is consistent with previous works in which N deficiency in *A. thaliana* was proven to affect galactolipid composition and result in the accumulation of fatty acid phytyl esters, including, among others, the α-linolenic acid phytyl ester [123]. Anthocyanins improve plant tolerance to low N; mutants with deficient biosynthesis show significantly lower survival rates when plants are grown under N-deprivation conditions [124].

Brassinosteroids interact with other phytohormones to stimulate antioxidant production and activate stress responsive transcription factors [68,69]. Diterpenoids, particularly gibberellins, act as protective molecules against oxidative stress and mediate plant growth [70,72]. Activating these pathways may increase the plant’s stress tolerance machinery under nitrate-deficient conditions, emphasizing a survival strategy focused on stress mitigation and growth regulation.

In contrast to rosettes, in the roots of the *nia1*/*nia2* double mutant, slightly more metabolic pathways were differentially enriched, especially at 4 mM. These enriched pathways include anthocyanin biosynthesis, steroid biosynthesis, sulfur metabolism, and folate biosynthesis (Figure 10c). In contrast, the phenylpropanoid biosynthesis pathway was the only enriched metabolic pathway under the N-deprivation condition (0.2 mM) (Figure 10d). Anthocyanin biosynthesis, which is enriched by antioxidant metabolites such as pelargonidin 3-O-glucoside and pelargonin, probably counteracts oxidative stress [72]. This interpretation is consistent with the role of anthocyanins in neutralizing reactive oxygen species and preventing oxidative damage. Concerning steroid biosynthesis, metabolites such as Δ7-avenasterol, 24-methylenephenol, 4α-methyl fecosterol, and stigmasterol play crucial roles in root elongation, growth, elasticity, and adaptation to abiotic stress [73,74]. The activation of sulfur metabolism (with sulfite) and folate biosynthesis (with metabolites such as 7,8-dihydropteroic acid, dihydropteroate, and 2,5-diamino-6-(5’-phosphoribosyl amino)-4-pyrimidinone) is also of particular interest. Folate biosynthesis is involved in redox homeostasis and has been shown to improve resistance to abiotic stress under nitrogen supplementation [75,76]. A link between S and N metabolism is known, with the deprivation of one disrupting the metabolism of the other [125,126]. In this crosstalk, O-acetyl-L-serine (OAS), the direct precursor of cysteine synthesis, is a key mediator located at the convergence of the S and N assimilation pathways, and its concentration changes in response to the N/S ratio in the growth medium [127]. Evidence suggests that many metabolic pathways are either N- or S-dependently connected [128,129,130,131], e.g., the early response to S deprivation caused a decrease in cysteine and glutathione (GSH) and the accumulation of precursors OAS and serine. The increase in serine is channeled to tryptophan, which leads to an increase in auxin, probably via indole glucosinolate catabolism, which may trigger root elongation to increase the accessibility of exogenous S (or N; reviewed in [132]). Finally, metabolites that enrich the phenylpropanoid biosynthesis pathway at 0.2 mM, including syringin, coniferin, and sinapyl alcohol, may also be involved in countering/alleviating the oxidative stress induced by increased ROS production [47,48,49].

**Figure 12:**
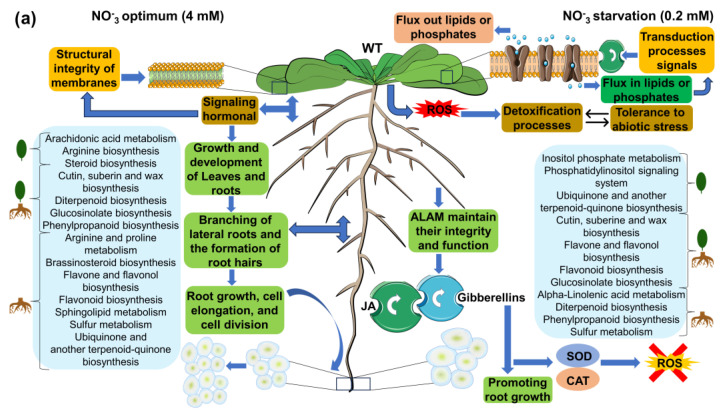

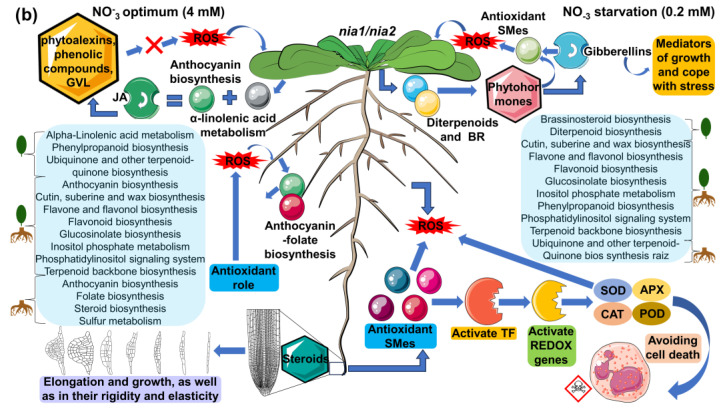
Diagram for the metabolomic response in the *A. thaliana* (**a**) WT and (**b**) loss-of-function *nia1*/*nia2* double mutant genotypes triggered by N-starvation. The metabolic responses in shoots and roots and summarized for both genotypes and under tested conditions, i.e., optimal (4 mM) and nitrate deficiency (0.2 mM) conditions. The differentially synthesized/accumulated metabolites help cope with stress, regulating oxidative stress and avoiding programmed cell death by apoptosis. As such, plants compromise growth to prioritize a defensive response.

Following a meticulous review and analysis of our experimental findings, an integrative model was constructed (Figure 12a,b) that not only synthesizes the most prominent results, but also weaves together the multiple facets of discussions stemming from this research.

## 5. Conclusions

The metabolomes analyzed from the *nia1*/*nia2* double mutant and WT genotypes suggest that under N-starvation conditions, plants prioritize survival over growth, meaning that when N availability is low, metabolites from distinct metabolic pathways that can help to counter the stress are synthesized (e.g., anthocyanins, phosphatidylinositol, or other forms of inositol, among others), whereas the synthesis of other metabolites that can promote plant growth and development (e.g., gibberellins or brassinosteroids) is arrested. The plasticity in the metabolomic responses enables a balance between growth and stress mitigation, representing a survival strategy when the environment is unfavorable. The results also suggest that a quick increase in ROS (oxidative burst) occurs when plants are under stress caused by N-deprivation. ROS triggers oxidative stress responses, which persist under conditions of sustained N-deprivation. Several compounds in the pathways show distinct levels of synthesis/accumulation in response to stress and depending on the organs being analyzed (e.g., the rosettes or roots). The difference may obey the distinct functions of both organs, i.e., roots attach the plant to a substrate for water and nutrient uptake (including N), whereas photosynthesis takes place inside the chloroplasts located in the mesophyll of the leaves (rosettes).

Although in the *nia1*/*nia2* double mutant and the WT genotypes, the N starvation responses are “comparable” at both phenotypic and metabolic levels, we did observe some slight differences. For example, seedlings grown at 4 mM were slightly larger than those grown at 0.2 mM. Although most of the enriched metabolic pathways were shared (at either 4 or 0.2 mM), a few were enriched mainly when seedlings were grown at an optimal N concentration (4 mM). We suggest that these differences may have occurred because the *nia1*/*nia2* double mutant cannot reduce nitrates, whereas the WT can directly absorb ammonium.

Finally, as in previous studies, we proved that some technical aspects, such as the preferential use of a unique ionization mode (either positive or negative), can generate biases, i.e., the metabolomes could be erroneously considered more diverse or richer in terms of the compounds they contain depending on the ESI mode used. Therefore, when distinct metabolomes, e.g., originating from different organs, conditions, or genotypes, are compared, the mass spectral libraries generated in both ESI modes correspond to the preferred representations of their compound compositions.

## Data Availability

All data generated or analyzed during this study are included in this published article (and its Supplementary Information Files).

## Supplementary Materials

The following supporting information can be downloaded at: https://www.mdpi.com/article/10.3390/metabo13091021/s1, Figure S1. Design of the time course experiment. (**a**) Using a hydroponic system, *A. thaliana* seedlings of two different genotypes (WT and *nia1*/*nia2* double mutant) were grown for 30 days (days after germination; dag) in MS media supplemented with either 0.2 mM (N-deprivation condition; nutritional stress) or 4 mM (optimal N concentration). The sampling points selected to analyze phenotypical traits and perform the metabolomic studies were 15 and 30 dag. The rosettes (shoots) and roots were harvested separately. Six plants were germinated in each hydroponic chamber, which was ordered as shown in (**b**) panel. Three biological replicates were made for each treatment. Figure S2. Seedling growth under aseptic and controlled conditions. The hydroponic chambers in which *A. thaliana* seedlings were sown were accommodated into a growth chamber with fluorescent light, constant temperature (22 °C) and using a photoperiod of 16 h light/8 h dark (see methods for details). Figure S3. Phenotypical traits of *Arabidopsis thaliana* seedlings. Seedlings were harvested from the hydroponic systems. The rosettes (shoots) and roots from both genotypes (WT and *nia/nia2* double) are shown at (**a**) 15 dag and (**b**) 30 dag. Figure S4. View of root growth into the hydroponic chambers. Elongation and growth of roots from the WT and the *nia1*/*nia2* double mutant genotypes at (**a**) 15 dag and (**b**) 30 dag. Roots’ growth at both N availability conditions (4 mM and 0.2 mM) are shown. Figure S5. Range-abundance curves of m/z_rt. A range-abundance curve is shown from each mass spectral library, considering both ESI modes independently. (**a**) ESI^+^ and (**b**) ESI^−^. Range-abundance curves show the position of/relevance of richness and accumulation of m/z_rt in the null model, indicating that the distribution of m/z_rt is modulated by N availability and shared uniformly by the m/z_rt. Figure S6. Analysis of variance (one-way ANOVA) of the *A. thaliana* metabolome obtained from each ESI mode. (**a**) and (**b**) show, for the ESI^+^ mode, the differences between the sampling points (15 and 30 dag) and N concentrations (4 and 0.2 mM), respectively. Same, but from ESI^−^ mode is shown in (**c**) and (**d**) panels. The boxplot graphs represent the mean of the data, the dispersion, and the interquartile range delimited by quartiles 1 (Q1) and 3 (Q3). The small letters above the boxplots represent Tukey’s *post hoc* test clustering. Figure S7. Pearson correlation and covariance between the Pielou (J’) and diversity. ESI modes were analyzed independently. Panels (**a**–**d**) correspond to ESI^+^ mode, while (**e**–**g**) represent ESI^−^ mode. In both ESI modes, obtaining positive correlative and covariance values stands out. The lines in mustard and green color correspond to the linear model of the correlation for each variable. The black line corresponds to the optimal theoretical linear distribution model, and the shadow in grey to its confidence intervals (95%). Figure S8. Spectral libraries’ normalization. For both ionization modes, (**a**) ESI^+^ and (**b**) ESI^−^, box plots show the range of intensities detected from each m/z_rt. The intensities’ distribution is shown for both raw data (left), and once they were normalized by quantile and log transformation (right). A maximum of 50 m/z_rt was shown due to space limitations in the graph. Besides, kernel density plots are also shown at the tops of box plots. Notice that once normalized, the intensities range shows a normal distribution. Figure S9. Principal component analysis (PCA). The explanatory percentage of variance values is shown for (**a**) the ESI^+^ mode and (**b**) the ESI^−^ mode. The first principal component (PC1) has the highest variance, followed by the second principal component (PC2). Figure S10. PCA bi-plot of the *A. thaliana* metabolome in both ionization modes. (**a**) Representation of the eigen values and eigen vectors obtained from the 2D PCA analysis in the ESI^+^ ionization mode. (**b**) Representation of the eigen values and eigen vectors obtained from the 2D PCA analysis in the ESI^−^ ionization mode. Figure S11. Heatmaps of the metabolomics dataset. The colors represent changes of each m/z_rt relative to the mean control level. Samples (*x*-axis) and m/z_rt (*y*-axis) are separated using hierarchical clustering (Ward’s algorithm), with the dendrogram being scaled to represent the distance between each branch (distance measure: Pearson’s correlation). (**a**) Show those genes identified in mass spectral libraries obtained in ESI^+^ mode; while (**b**) show the same for ESI^−^ mode. Figure S12. Decision trees (regression trees) and the variable importance measures (genotypes and N concentrations) from sampling points. (**a**) illustrates a regression tree from the dataset obtained in ESI^+^ mode. On the first split, the decision tree separates nitrate concentrations from genotypes. The tree is pruned to the size that has the lowest cross-validation error. At each terminal node, the average relative abundance of m/z_rt allocated to the node is shown. (**b**) panel show the same but from the dataset obtained in ESI^−^ mode. Figure S13. Volcano plots showing the differentially synthesized/accumulated m/z_rt. According to pairwise comparisons performed (Table S9), differentially synthesized/accumulated m/z_rt were identified. (**a**) illustrate, as an example, the differentially synthesized/accumulated m/z_rt identified in ESI^+^ mode from the contrast 15dag_4mM_WT_rosette versus 15dag_0.2mM_WT_rosette. (**b**) Same contrast but from those differentially synthesized/accumulated m/z_rt identified in ESI^−^ mode. Datasets were filtered to remove m/z_rt with low relative abundance (dotted line from −1 to 1 on the *x*-axis), and a significance cut-off (*p*-value ≤ 0.01) was applied (dotted line on the *y*-axis). Figure S14. Bar plots summarizing the total of the differentially synthesized/accumulated m/z_rt. According to each pairwise comparison performed (*x*-axis, see details in Table S9), the total of differentially synthetized/accumulated m/z_rt (*y*-axis) is shown. The number of differentially synthesized/accumulated m/z_rt is ordered from smallest to largest and is shown independently for both ESI modes. (**a**) ESI^+^ and (**b**) ESI^−^. Figure S15. Reconstruction of metabolic pathways of the metabolome of *A. thaliana*. The six main and mostly enriched metabolic pathways corresponding to the secondary metabolism of *A. thaliana* are shown in a representative way. Within each metabolic pathway, the points marked in yellow represent those metabolites identified in mass spectral libraries and with a presumptive annotation by KEGG code. The color scale alludes to the presence/absence of the SMe within the pathway. (**a**) Cutin, suberin and wax biosynthesis, (**b**) flavone and flavonol biosynthesis, (**c**) flavonoid biosynthesis, (**d**) glucosinolate biosynthesis, (**e**) phenylalanine, tyrosine and tryptophan biosynthesis, (**f**) phenylpropanoid biosynthesis. Table S1. Concentrations of the culture medium of Murashige and Skoog (MS). Table S2. Supplements used in the Murashige and Skoog (MS) culture medium for the hydroponic growing of *A. thaliana* seedlings. Table S3. Pairs of samples/treatments compared (pairwise comparison) to identify differentially synthesized/accumulated m/z_rt. First, (**a**) considering both ESI modes independently; then, (**b**) once they were merged, just like data from both sampling points (15 and 30 dag) were merged. In the above, in the occurrence of variance, the N availability and the genotypes were the most critical variables (Figure S11). Table S4. Charge ratio values (m/z) from the mass spectral libraries obtained in the ESI^+^ mode. Table S5. Charge ratio values (m/z) from the mass spectral libraries obtained in the ESI^−^ mode. Table S6. Shannon–Wiener (H’), Simpson (DSi) α diversity, equity of Pielou (J’) values obtained from the “species” (m/z_rt) on each sampling site (treatments). These estimators were calculated using the mass spectral libraries obtained in ESI^+^ mode. Table S7. Shannon–Wiener (H’), Simpson (DSi) α diversity, equity of Pielou (J’) values obtained from the “species” (m/z_rt) on each sampling site (treatments). These estimators were calculated using the mass spectral libraries obtained in ESI^−^ mode. Table S8. Principal Component Analysis (PCA) results in which the entire dataset from ESI^+^ mode was included and shows the correlation coefficients for the total variables included on the distinct components (48 in total) and for 1635 m/z_rt. In the rows, numbers indicate the correlation of each m/z_rt with the eigenvector of each PC. Table S9. Principal Component Analysis (PCA) results in which the entire dataset from ESI^−^ mode was included and shows the correlation coefficients for the total variables included on the distinct components (48 in total) and for 1470 m/z_rt. In the rows, numbers indicate the correlation of each m/z_rt with the eigenvector of each PC. Table S10. Differentially synthesized/accumulated m/z_rt identified by fold-change (FC) analyses. ESI modes were considered separately, and both (positive and negative) are shown—columns B to CB from ESI^+^ and CF to FF from ESI^−^. Table S11. Presumptive annotation of differentially synthesized/accumulated m/z_rt and their metabolic pathways. Table S12. Presumptive metabolites (annotated m/z_rt) and their metabolic pathways. From left to right, and for each presumptive metabolite, name, formula, the mass-to-charge ratio (m/z), retention time (rt), KEGG code, and ESI mode in which they were detected are shown.
