# Supplementary material for: Non-Targeted Metabolomic Analysis of Arabidopsis thaliana (L.) Heynh: Metabolic Adaptive Responses to Stress Caused by N Starvation
Source: Metabolites. 2023 Sep 18;13(9):1021. doi: 10.3390/metabo13091021 (PMC10535036; doi:10.3390/metabo13091021)
Supplement: Supplementary file 1 [file metabolites-13-01021-s001.zip › SupplementaryFiles/Supplementary Figures (S1-S15).pdf]

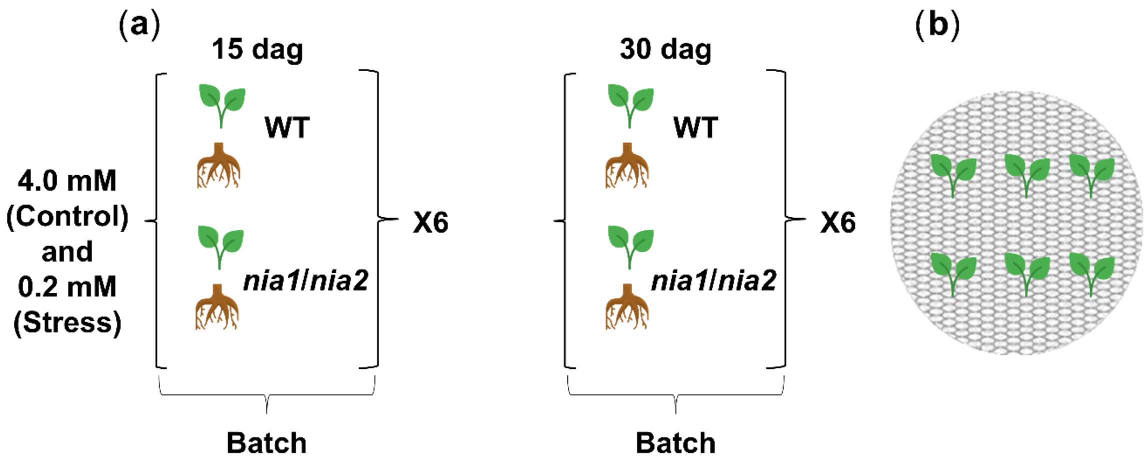

Figure S1. Design of the time-course experiment. (**a**) Using a hydroponic system, *A. thaliana* seedlings of two different genotypes (WT and *nia1/nia2* double mutant) were grown for 30 days (days after germination; dag) in MS media supplemented with either 0.2 mM (N deprivation condition; nutritional stress) or 4 mM (optimal N concentration). The sampling points selected to analyze phenotypical traits and perform the metabolomic studies were 15 and 30 dag. The rosettes (shoots) and roots were harvested separately. Six plants were germinated in each hydroponic chamber, which was ordered as shown in (**b**) panel. Three biological replicates were made for each treatment.

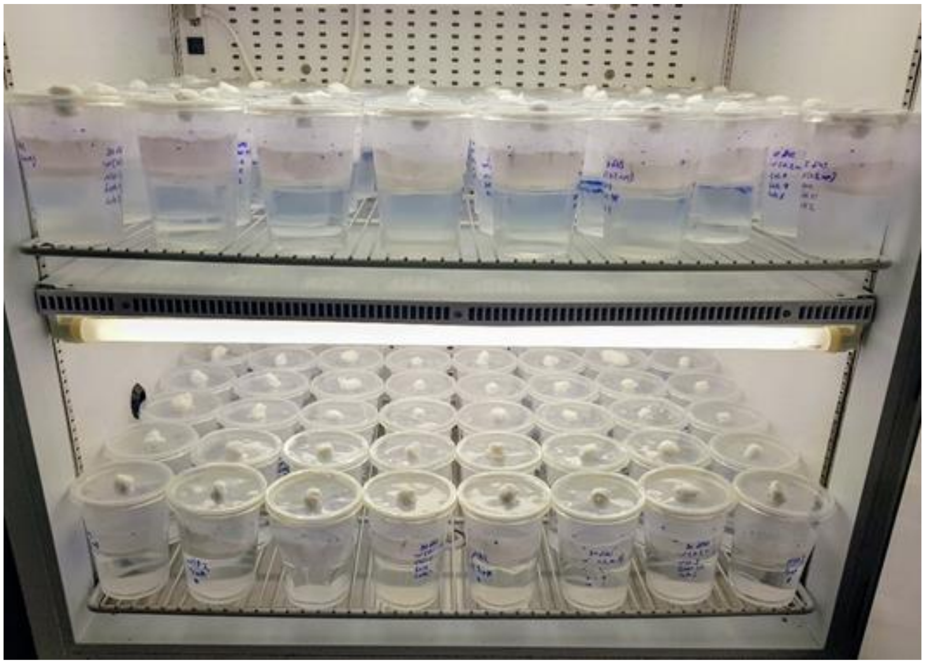

Figure S2. Seedling growth under aseptic and controlled conditions. The hydroponic chambers in which *A. thaliana* seedlings were sown were accommodated into a growth chamber with fluorescent light, constant temperature (22 °C) and using a photoperiod of 16 h light/8 h dark (see methods for details).

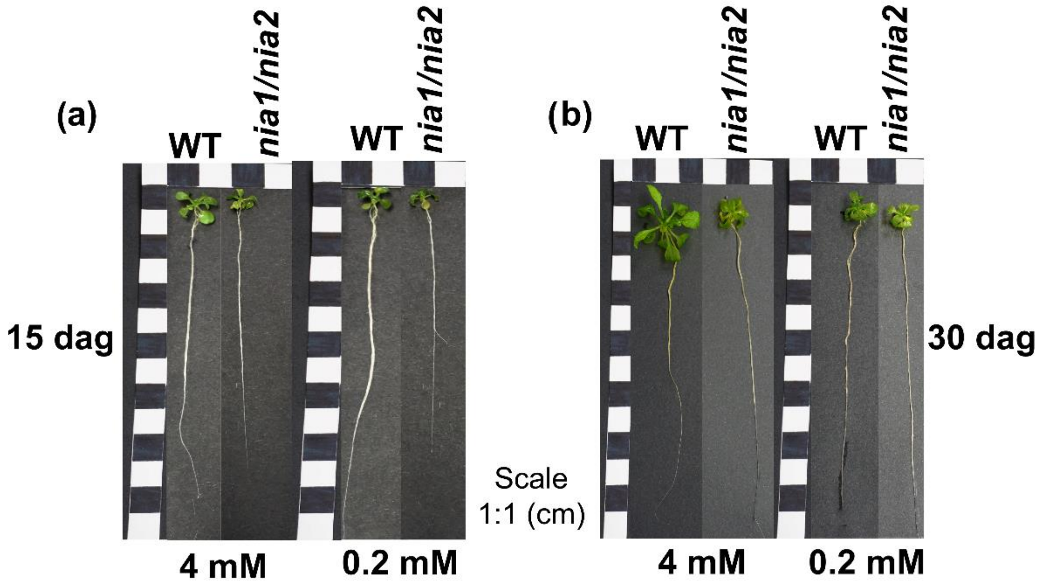

Figure S3. Phenotypical traits of *Arabidopsis thaliana* seedlings. Seedlings were harvested from the hydroponic systems. The rosettes (shoots) and roots from both genotypes (WT and *nia/nia2* double) are shown at (**a**) 15 dag and (**b**) 30 dag.

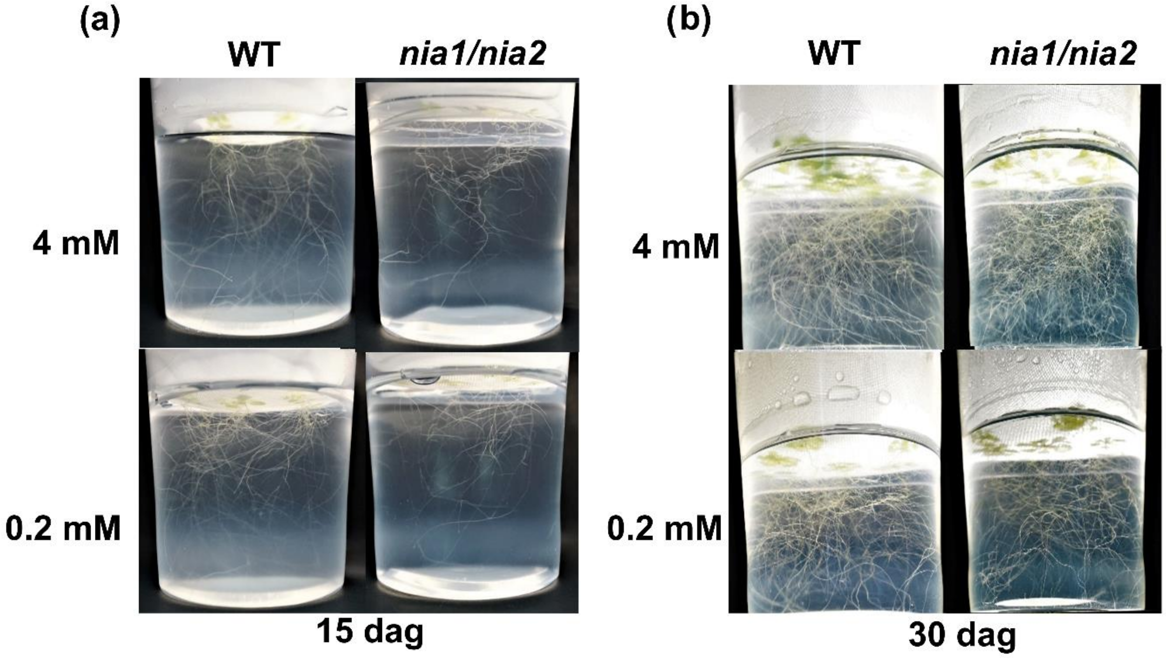

Figure S4. View of roots growth into the hydroponic chambers. Elongation and growth of roots from the WT and the *nia1/nia2* double mutant genotypes at (**a**) 15 dag and (**b**) 30 dag. Roots' growth at both N availability conditions (4 mM and 0.2 mM) are shown.

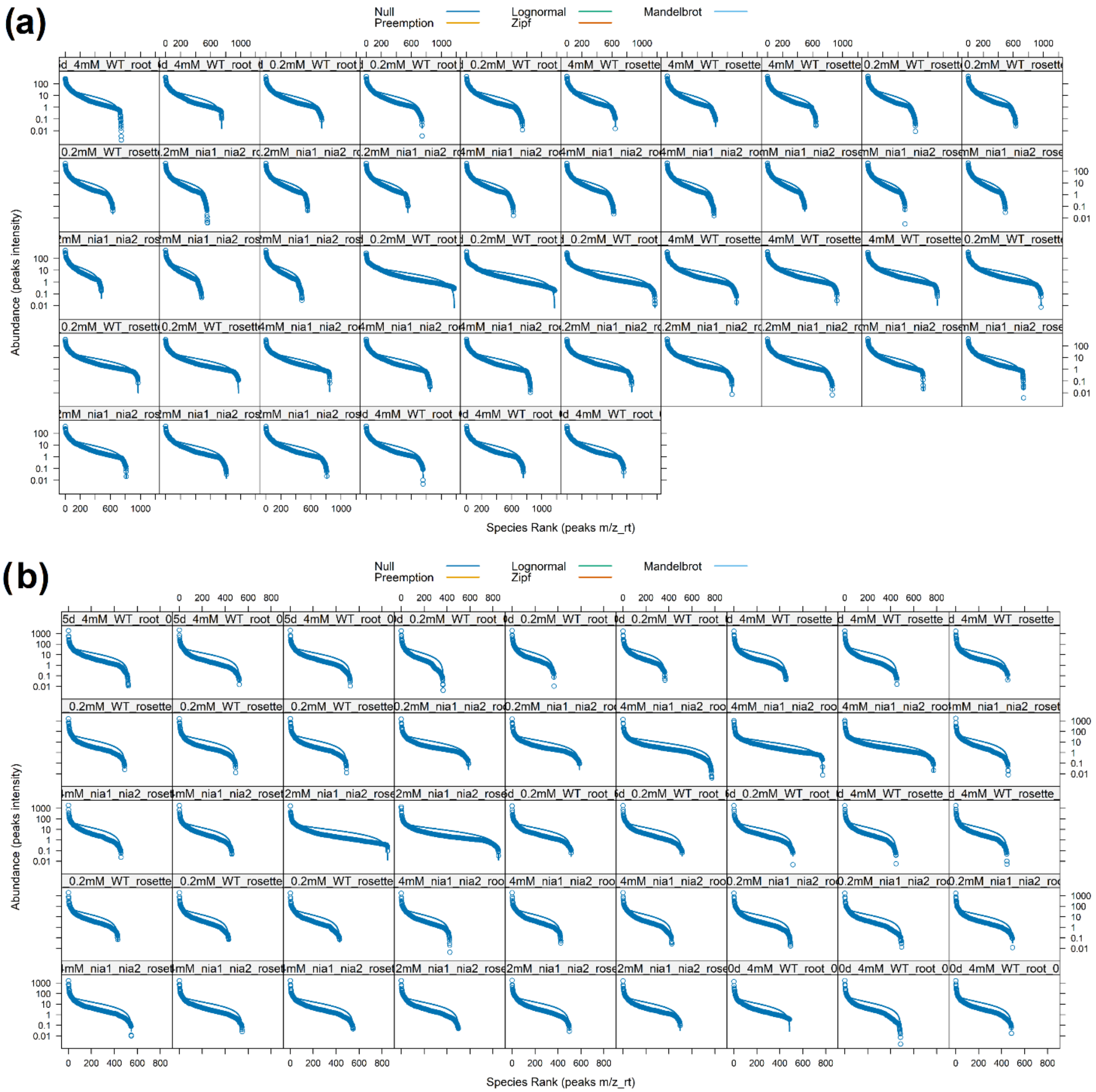

Figure S5. Range-abundance curves of m/z_rt. A range-abundance curve is shown from each mass spectral library, considering both ESI modes independently. (**a**) ESI^+ and (**b**) ESI^-. Range-abundance curves show the position of / relevance of richness and accumulation of m/z_rt in the null model, indicating that the distribution of m/z_rt is modulated by N availability and shared uniformly by the m/z_rt.

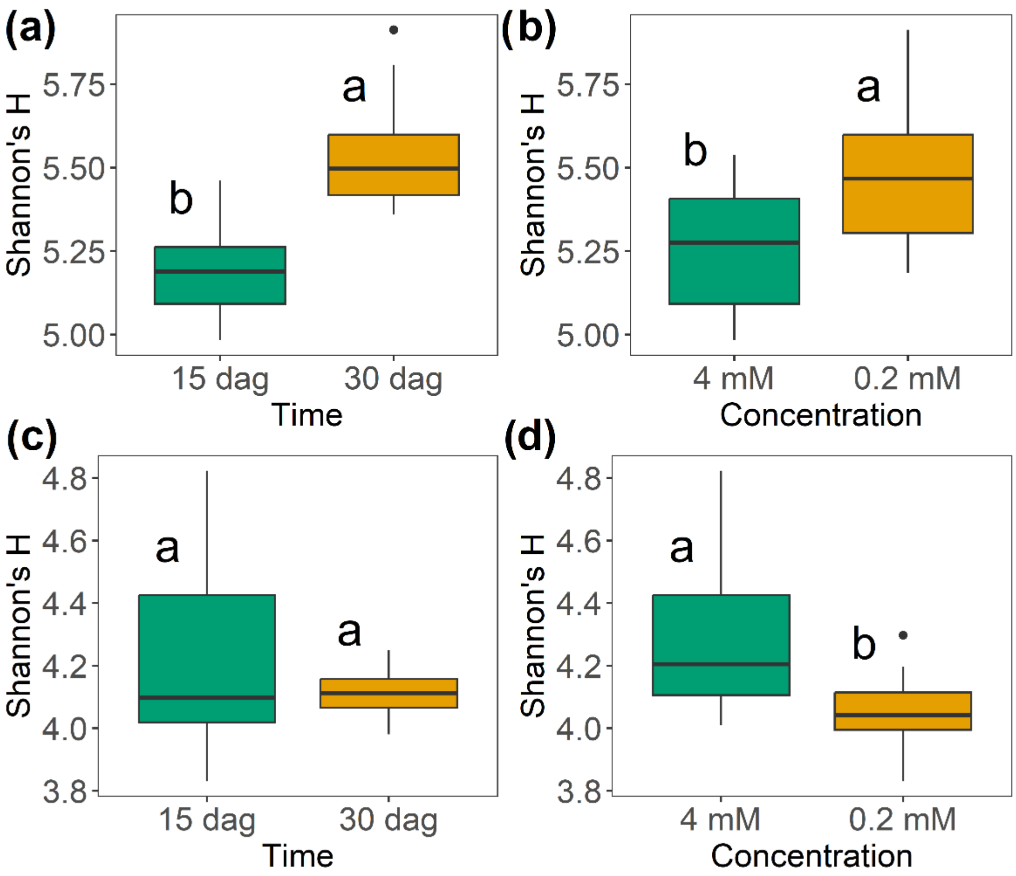

Figure S6. Analysis of variance (one-way ANOVA) of the *A. thaliana* metabolome obtained from each ESI mode. (**a**) and (**b**) show, for the ESI^+ mode, the differences between the sampling points (15 and 30 dag) and N concentrations (4 and 0.2 mM), respectively. Same, but from ESI^- mode is shown in (**c**) and (**d**) panels. The boxplot graphs represent the mean of the data, the dispersion, and the interquartile range delimited by quartiles 1 (Q1) and 3 (Q3). The small letters above the boxplots represent Tukey's *post-hoc* test clustering.

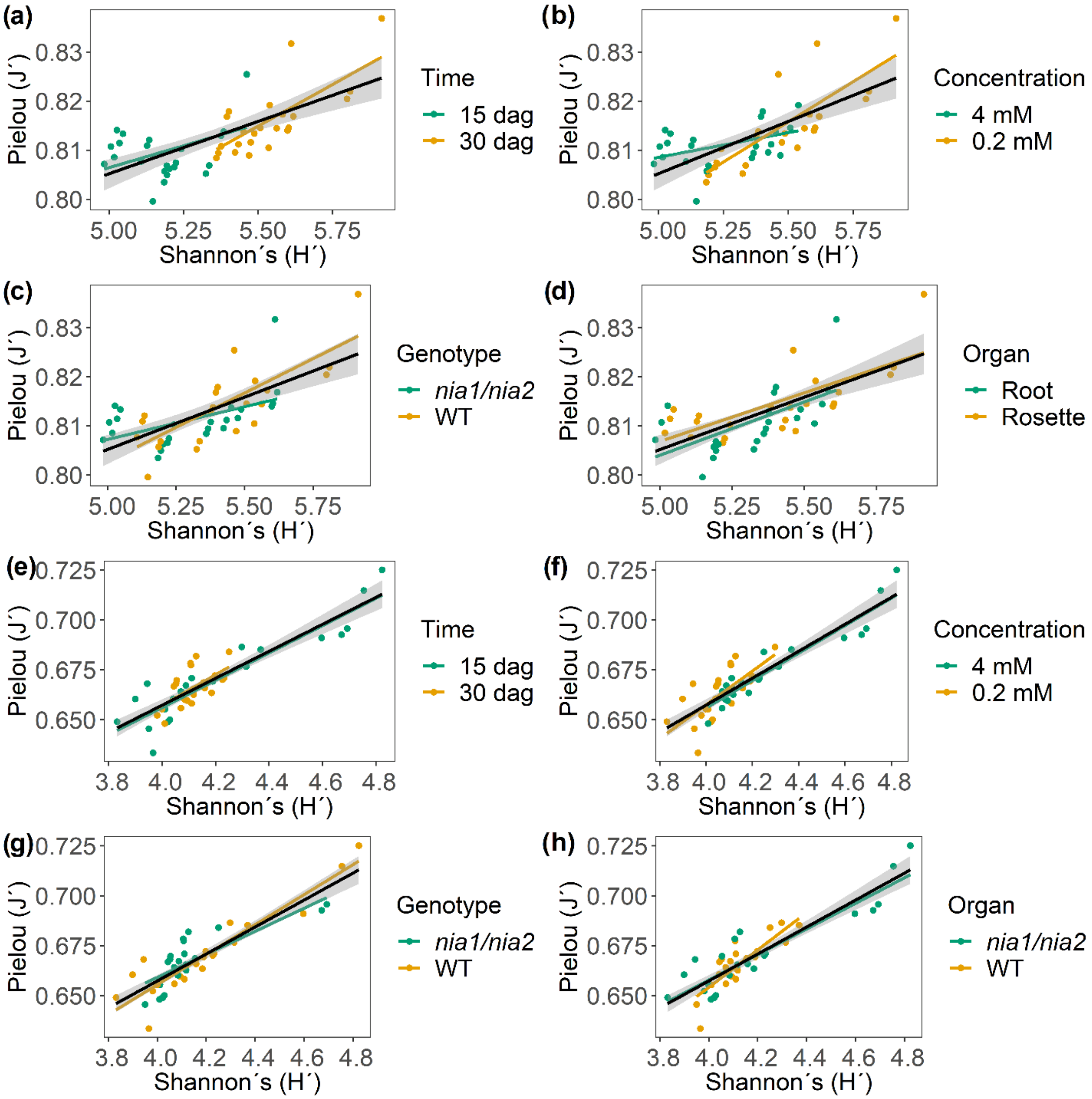

Figure S7. Pearson correlation and covariance between the Pielou (J') and diversity. ESI modes were analyzed independently. Panels (**a**), (**b**), (**c**), and (**d**) correspond to ESI+ mode, while (**e**), (**f**), (**g**), and (**h**) panels represent ESI- mode. In both ESI modes, obtaining positive correlative and covariance values stands out. The lines in mustard and green color correspond to the linear model of the correlation for each variable. The black line corresponds to the optimal theoretical linear distribution model, and the shadow in grey to its confidence intervals (95%).

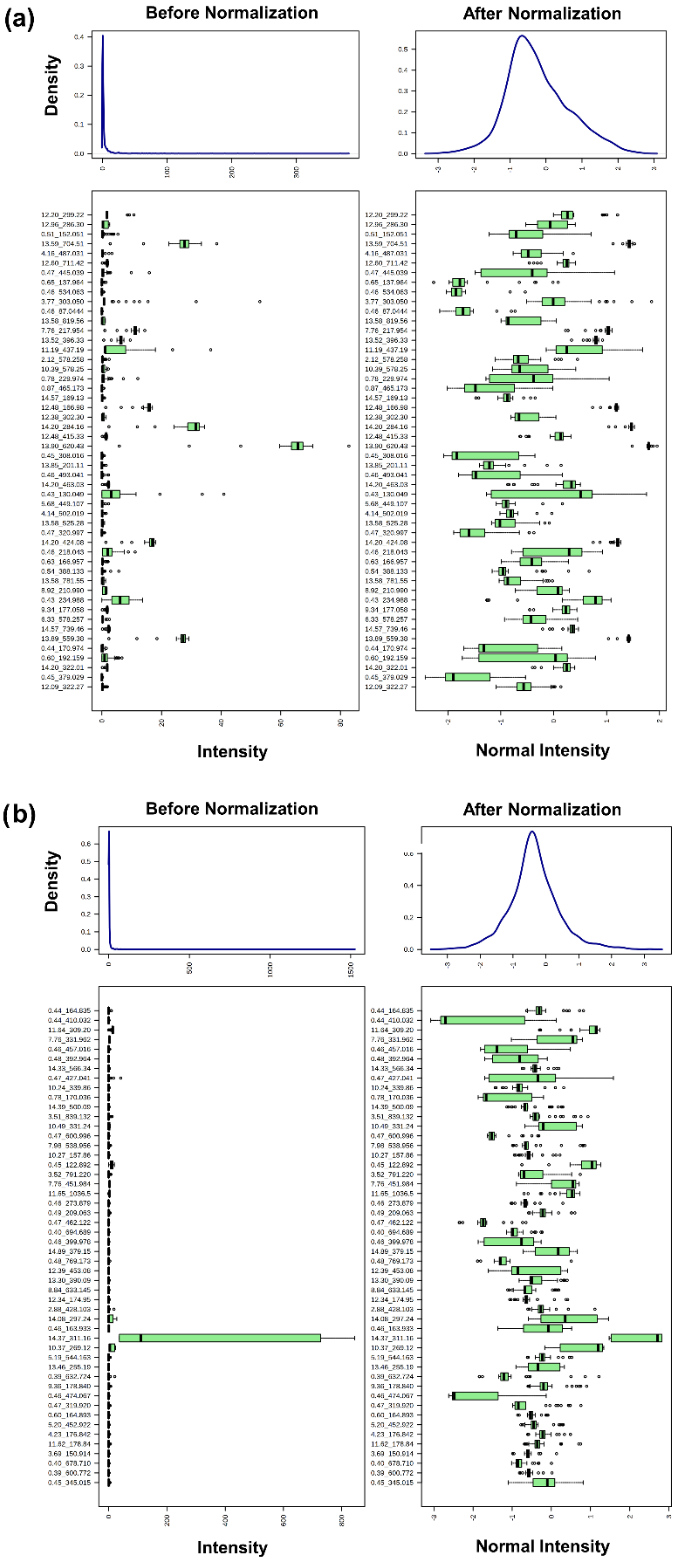

Figure S8. Spectral libraries normalization. For both ionization modes, (**a**) ESI^+ and (**b**) ESI^-, box plots show the range of intensities detected from each m/z_rt. The intensities distribution is shown for both raw data (left), and once they were normalized by quantile and log transformation (right). A maximum of 50 m/z_rt was shown due to space limitations in the graph. Besides, kernel density plots are also shown at the top of box plots. Notice that once normalized, the intensities range shows a normal distribution.

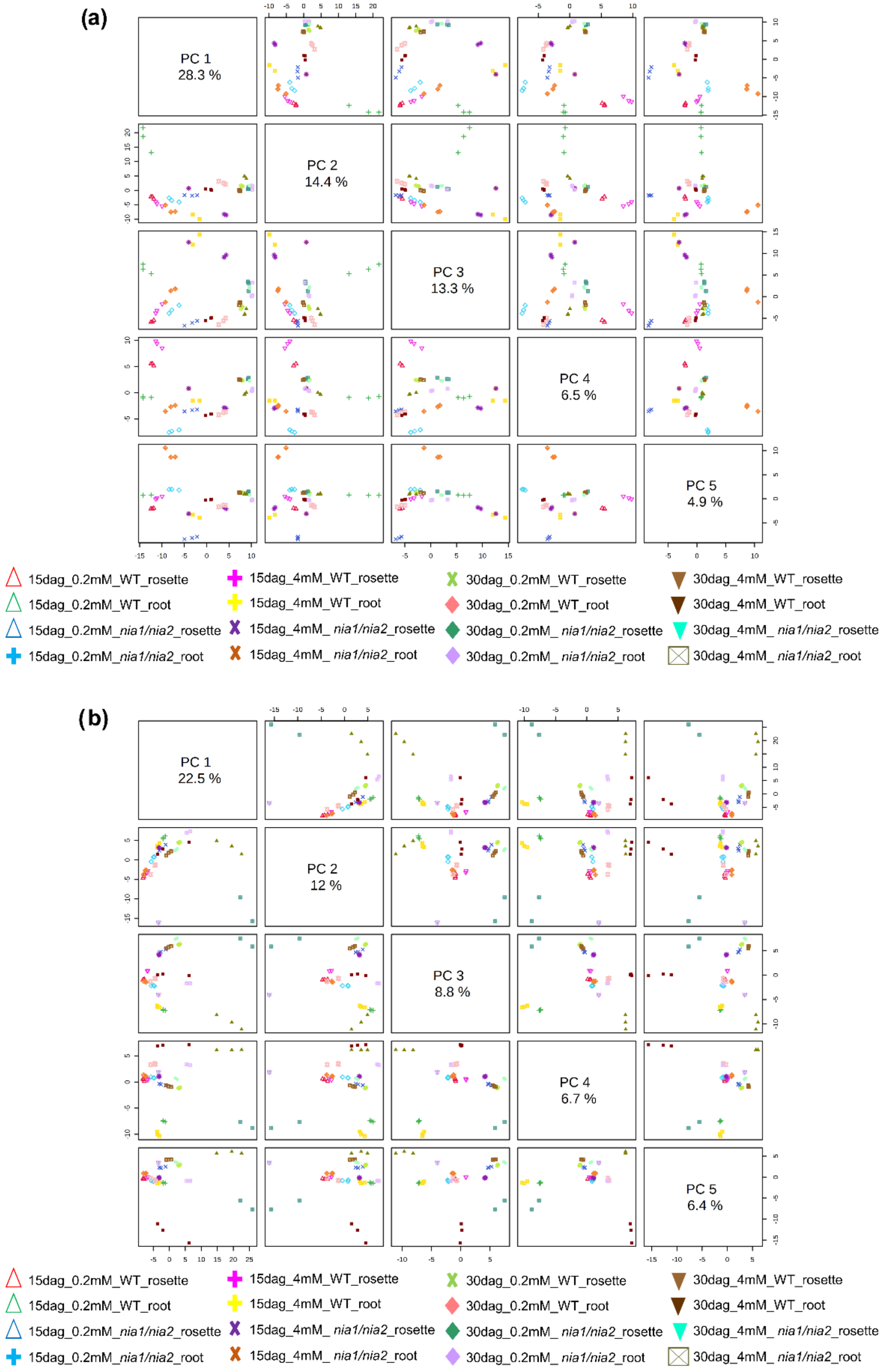

Figure S9. Principal component analysis (PCA). The explanatory percentage of variance values is shown for (**a**) the ESI$^+$ mode and (**b**) the ESI$^-$ mode. The first principal component (PC1) has the highest variance, followed by the second principal component (PC2).

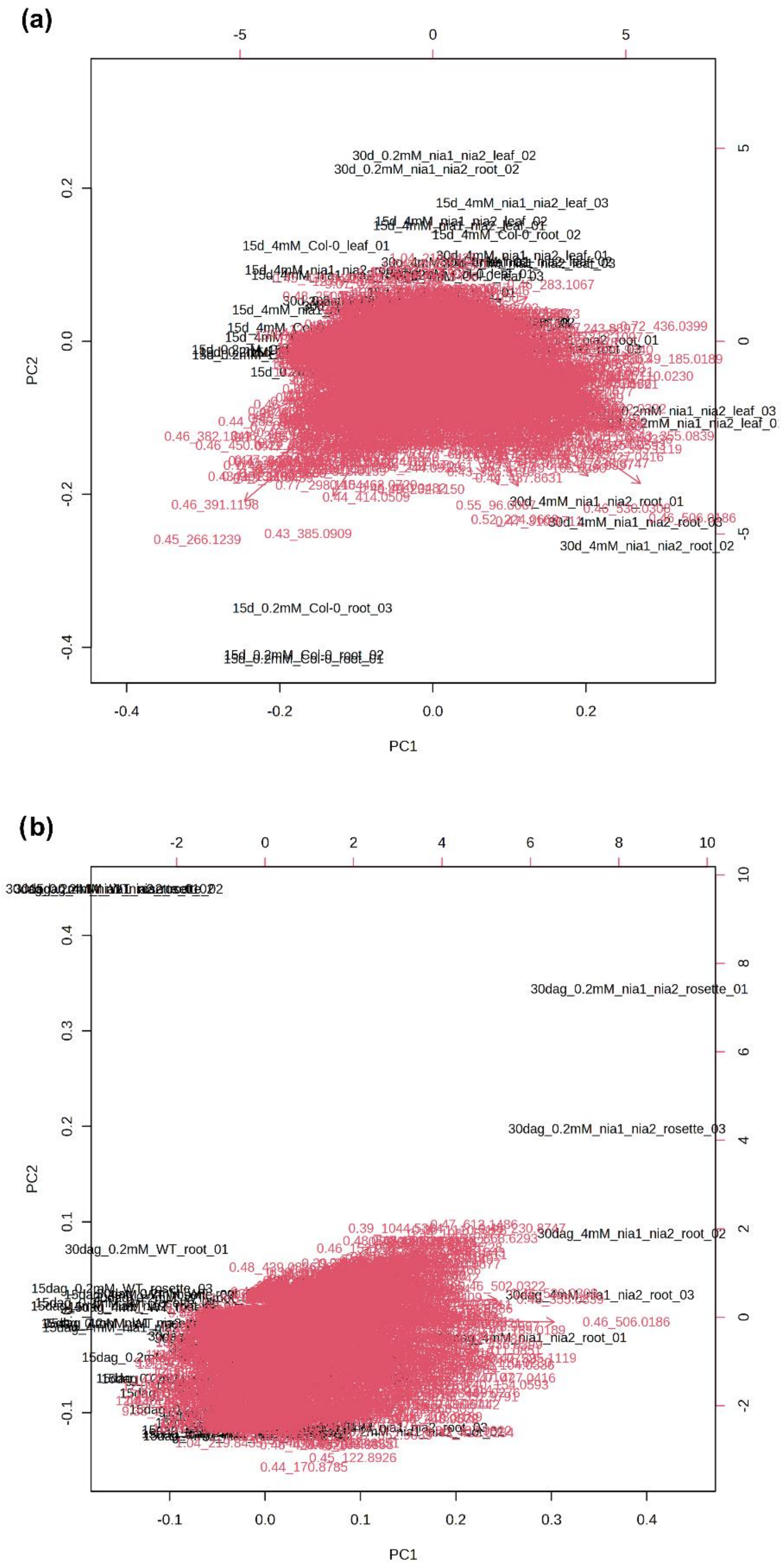

Figure S10. PCA bi-plot of the *A. thaliana* metabolome in both ionization modes. (**a**) Representation of the eigen values and eigen vectors obtained from the 2D PCA analysis in the ESI$^+$ ionization mode. (**b**) Representation of the eigen values and eigen vectors obtained from the 2D PCA analysis in the ESI$^-$ ionization mode.

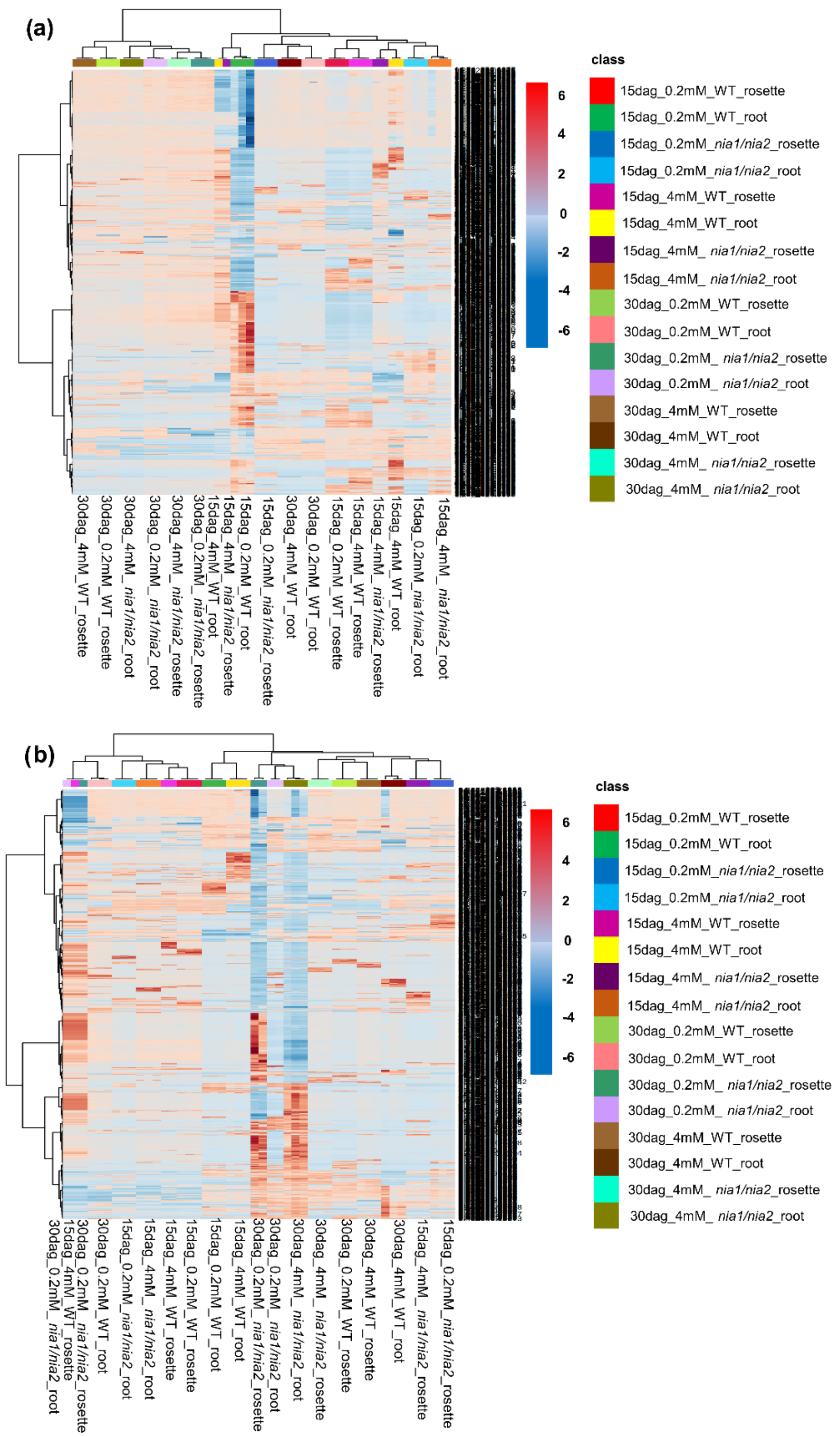

Figure S11. Heatmaps of the metabolomics dataset. The colors represent changes of each m/z_rt relative to the mean control level. Samples (*x*-axis) and m/z_rt (*y*-axis) are separated using hierarchical clus-tering (Ward's algorithm), with the dendrogram being scaled to represent the distance between each branch (distance measure: Pearson's correlation). (**a**) Show those genes identified in mass spectral libraries obtained in ESI$^+$ mode, while (**b**) show the same for ESI$^-$ mode.

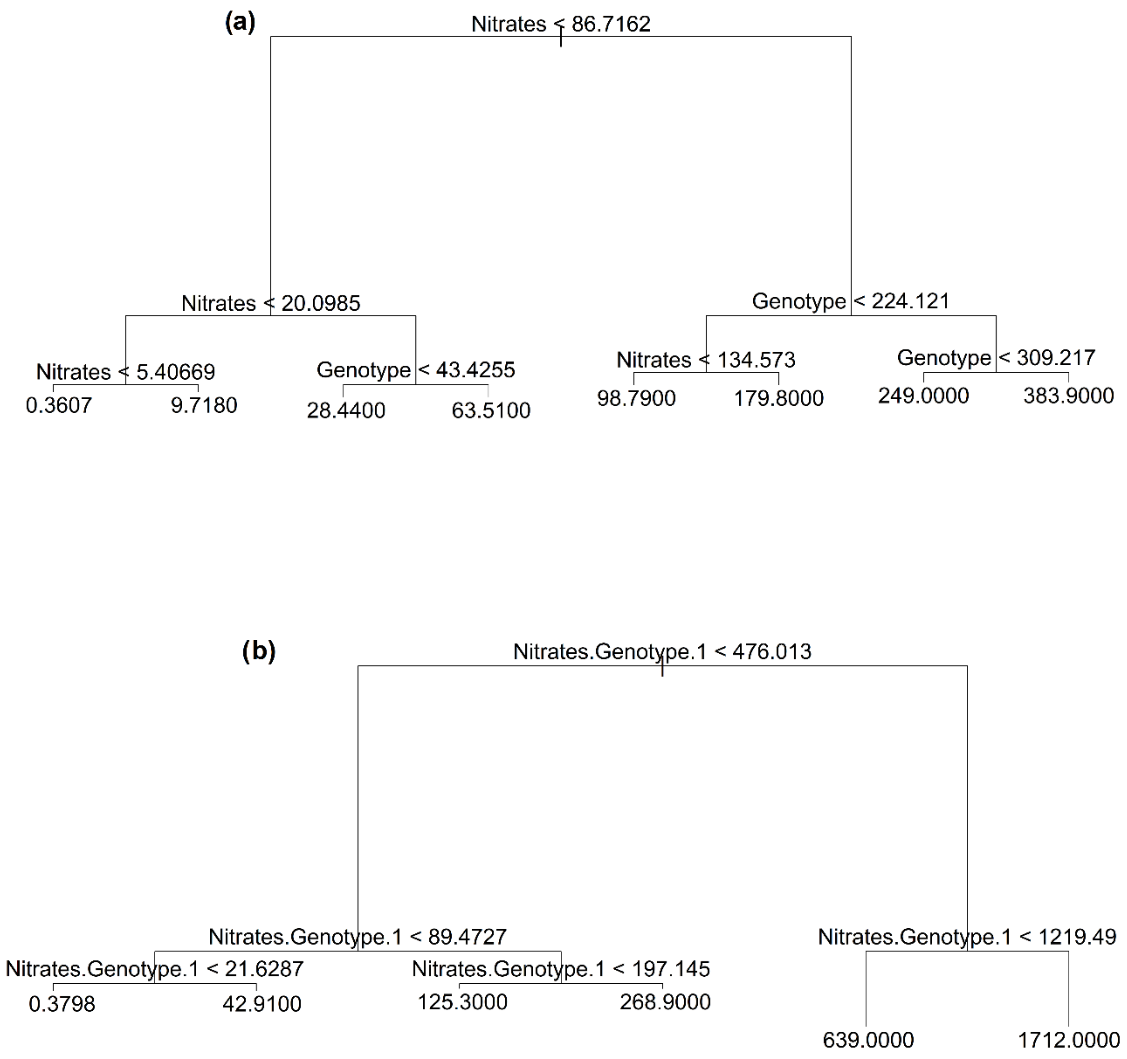

Figure S12. Decision trees (regression trees) and the variable importance measures (genotypes and N concentrations) from sampling points. (**a**) illustrates a regression tree from the dataset obtained in ESI^+ mode. On the first split, the decision tree separates nitrates concentrations from genotypes. The tree is pruned to the size that has the lowest cross-validation error. At each terminal node, the average relative abundance of m/z_rt allocated to the node is shown. (**b**) panel show the same but from the dataset obtained in ESI^- mode.

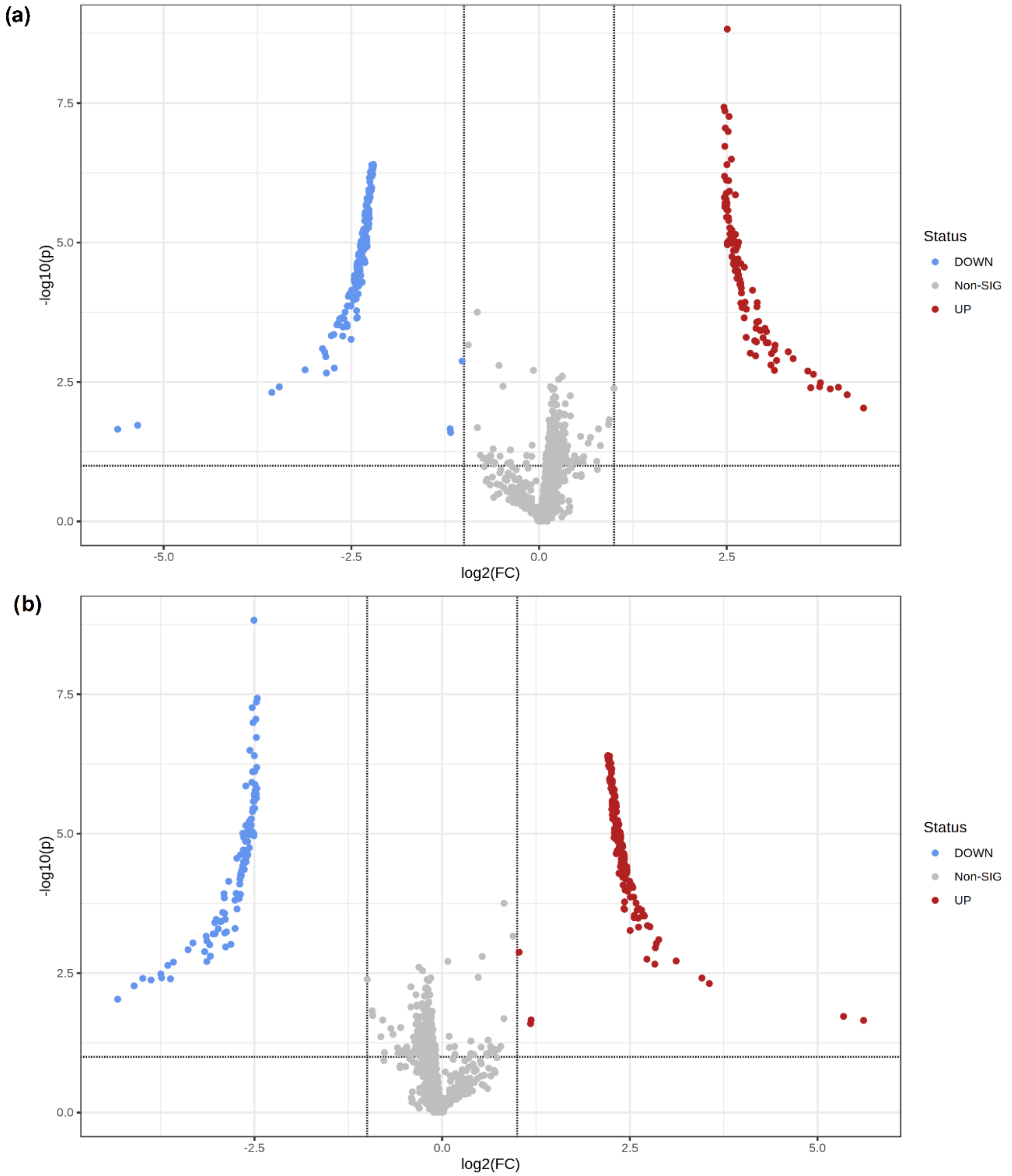

Figure S13. Volcano plots showing the differentially synthesized/accumulated m/z_rt. According to pairwise comparisons performed (Table S9), differentially synthesized/accumulated m/z_rt were identified. (**a**) illustrate, as an example, the differentially synthesized/accumulated m/z_rt identified in ESI^+ mode from the contrast 15dag_4mM_WT_rosette *versus* 15dag_0.2mM_WT_rosette. (**b**) Same contrast but from those differentially synthesized/accumulated m/z_rt identified in ESI^- mode. Datasets were filtered to remove m/z_rt with low relative abundance (dotted line from −1 to 1 on the *x*-axis), and a significance cut-off (p-value ≤ 0.01) was applied (dotted line on the *y*-axis).

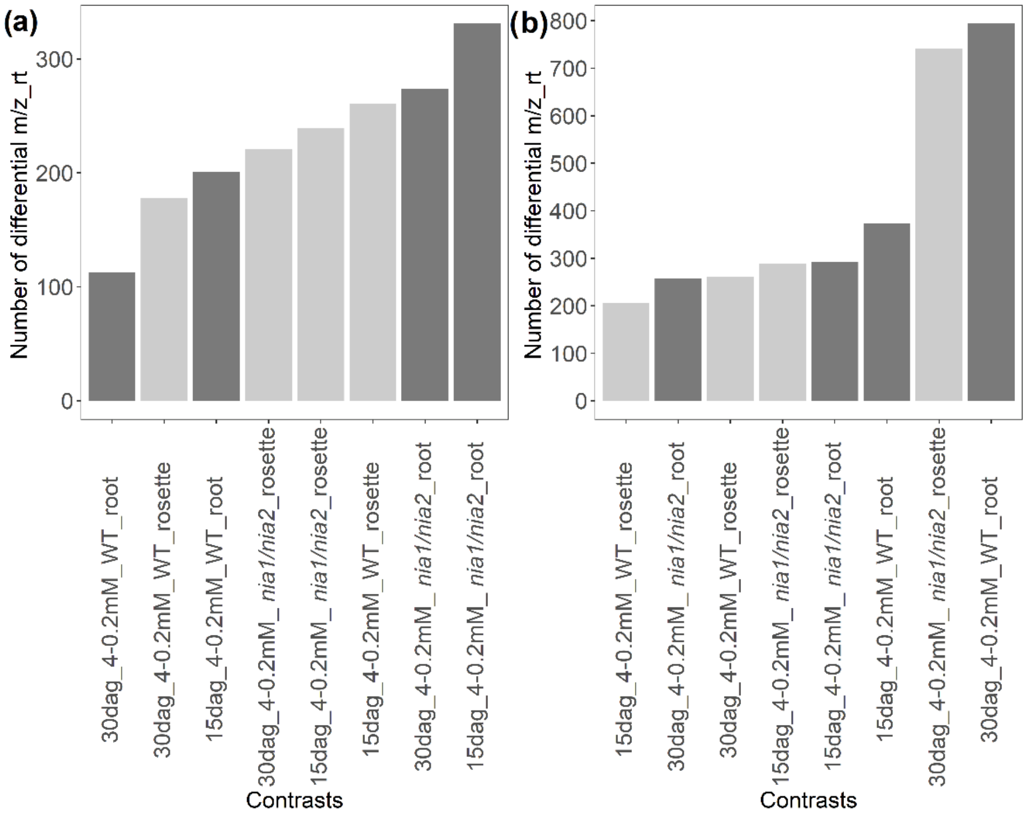

Figure S14. Bar plots summarizing the total of the differentially synthesized/accumulated m/z_rt. According to each pairwise comparison performed (*x*-axis, see details in Table S9), the total of differentially synthesized/accumulated m/z_rt (*y*-axis) is shown. The number of differentially synthetized/accumulated m/z_rt is ordered from smallest to largest and is shown independently for both ESI modes. (**a**) ESI^+ and (**b**) ESI^-.

(a)

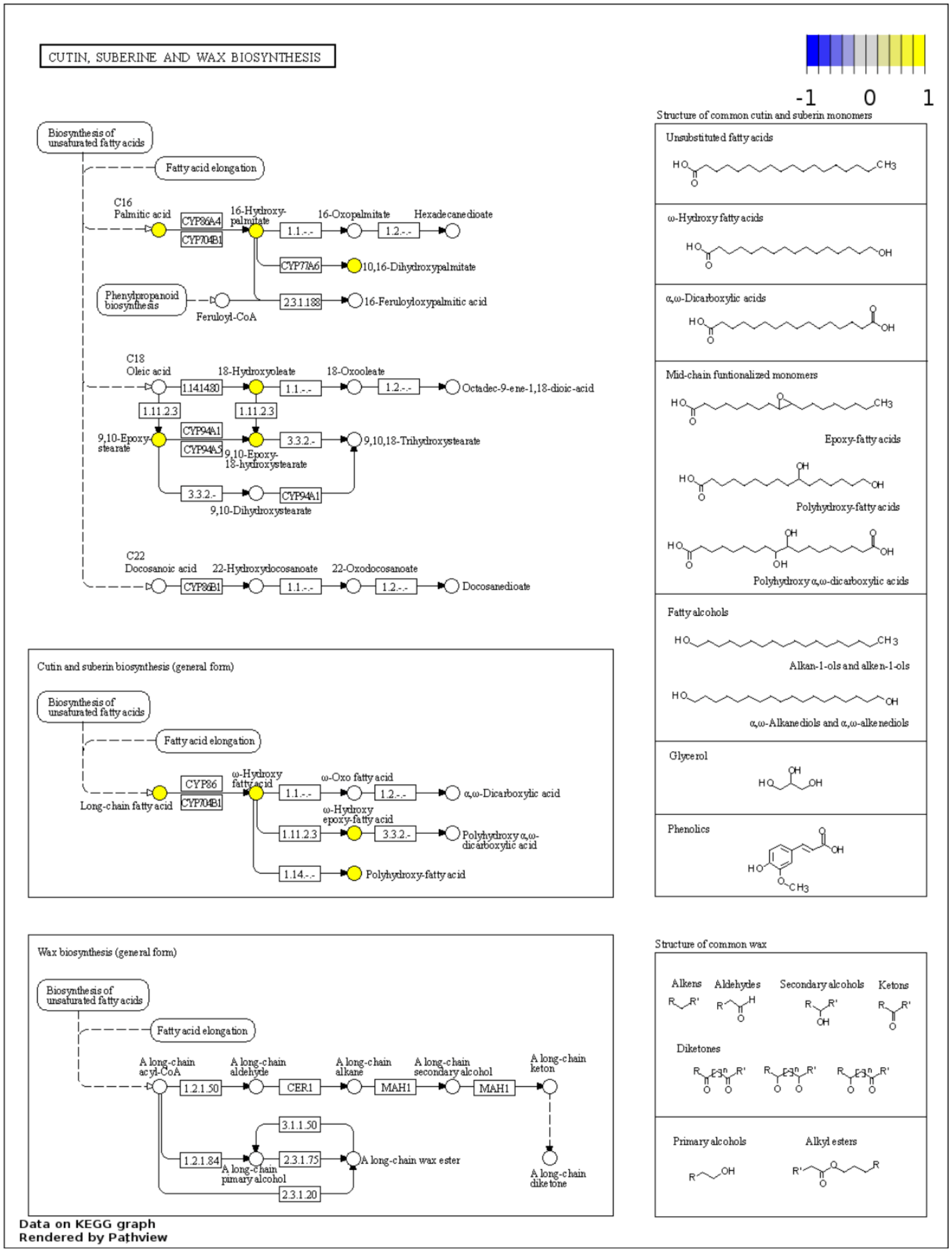
CUTIN, SUBERINE AND WAX BIOSYNTHESIS
-1 0 1
Structure of common cutin and suberin monomers
Unsubstituted fatty acids
ω-Hydroxy fatty acids
α,ω-Dicarboxylic acids
Mid-chain funtionalized monomers
Epoxy-fatty acids
Polyhydroxy-fatty acids
Polyhydroxy α,ω-dicarboxylic acids
Fatty alcohols
Alkan-1-ols and alken-1-ols
α,ω-Alkanediols and α,ω-alkenediols
Glycerol
Phenolics
Biosynthesis of unsaturated fatty acids
Fatty acid elongation
C16 Palmitic acid
CYP86A4
CYP704B1
16-Hydroxy-palmitate
1.1.-.-
16-Oxopalmitate
1.2.-.-
Hexadecanedioate
CYP77A6
10,16-Dihydroxypalmitate
Phenylpropanoid biosynthesis
Feruloyl-CoA
2.3.1.188
16-Feruloyloxypalmitic acid
C18 Oleic acid
1.14.14.80
18-Hydroxyoleate
18-Oxooleate
Octadec-9-ene-1,18-dioic-acid
1.11.2.3
9,10-Epoxy-stearate
CYP94A1
CYP94A5
9,10-Epoxy-18-hydroxystearate
3.3.2.-
9,10,18-Trihydroxystearate
9,10-Dihydroxystearate
C22 Docosanoic acid
CYP86B1
22-Hydroxydocosanoate
22-Oxodocosanoate
Docosanedioate
Cutin and suberin biosynthesis (general form)
Long-chain fatty acid
CYP86
ω-Hydroxy fatty acid
ω-Oxo fatty acid
α,ω-Dicarboxylic acid
ω-Hydroxy epoxy-fatty acid
Polyhydroxy α,ω-dicarboxylic acid
1.14.-.-
Polyhydroxy-fatty acid
Wax biosynthesis (general form)
A long-chain acyl-CoA
1.2.1.50
A long-chain aldehyde
CER1
A long-chain alkane
MAH1
A long-chain secondary alcohol
A long-chain keton
A long-chain diketone
3.1.1.50
1.2.1.84
2.3.1.75
A long-chain pumary alcohol
A long-chain wax ester
2.3.1.20
Structure of common wax
Alkens
Aldehydes
Secondary alcohols
Ketons
Diketones
Primary alcohols
Alkyl esters
Data on KEGG graph
Rendered by Pathview

(b)

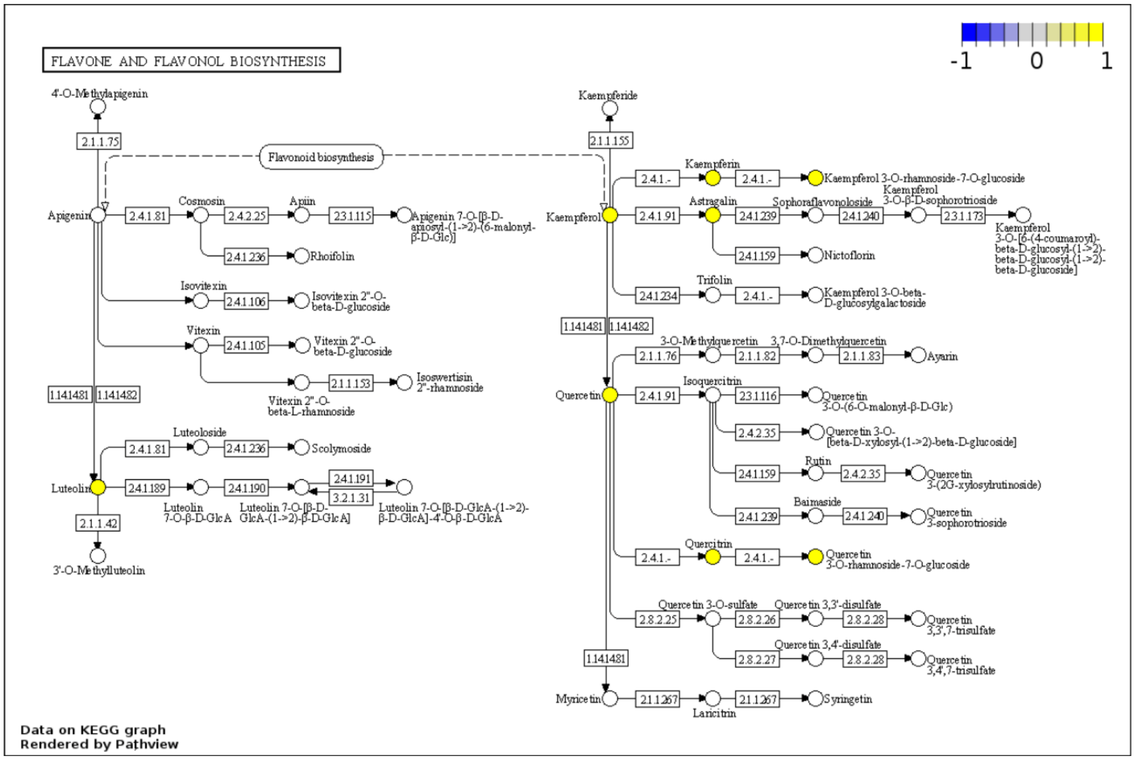
FLAVONE AND FLAVONOL BIOSYNTHESIS
-1
0
1
4'-O-Methylapigenin
Kaempferide
Flavonoid biosynthesis
Apigenin
Cosmosin
Apiin
Rhoifolin
Isovitexin
Vitexin
Kaempferol
Kaempferin
Astragalin
Sophoraflavonoloside
Nictoflorin
Trifolin
Quercetin
Isoquercitrin
Rutin
Baimaside
Quercitrin
Luteolin
Luteoloside
Scolymoside
3'-O-Methylluteolin
Myricetin
Laricitrin
Syringetin
Ayarin
Data on KEGG graph
Rendered by Pathview

(c)

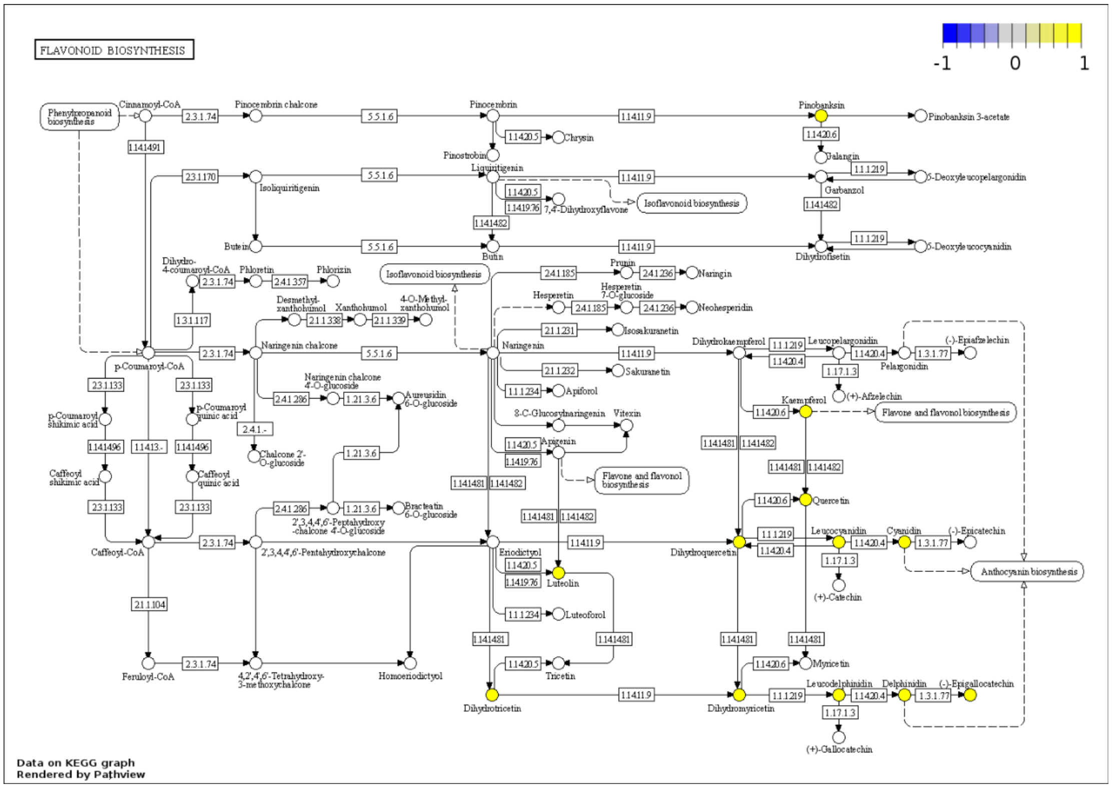
FLAVONOID BIOSYNTHESIS
-1
0
1
Phenylpropanoid biosynthesis
Cinnamoyl-CoA
Pinocembrin chalcone
Pinocembrin
Pinobanksin
Pinobanksin 3-acetate
Chrysin
Galangin
Liquiritigenin
Isoliquiritigenin
Garbanzol
Isoflavonoid biosynthesis
Butein
Butin
Dihydrofisetin
Naringenin chalcone
Naringenin
Naringin
Hesperetin
Neohesperidin
Isosakuranetin
Sakuranetin
Apiforol
Dihydrokaempferol
Leucopelargonidin
Pelargonidin
(-)-Epiafzelechin
(+)-Afzelechin
Kaempferol
Flavone and flavonol biosynthesis
Quercetin
Apigenin
Vitexin
p-Coumaroyl-CoA
Caffeoyl-CoA
Eriodictyol
Luteolin
Luteoforol
Dihydroquercetin
Leucocyanidin
Cyanidin
(-)-Epicatechin
(+)-Catechin
Anthocyanin biosynthesis
Feruloyl-CoA
Homoeriodictyol
Tricetin
Myricetin
Dihydrotricetin
Dihydromyricetin
Leucodelphinidin
Delphinidin
(-)-Epigallocatechin
(+)-Gallocatechin
Data on KEGG graph
Rendered by Pathview

(d)

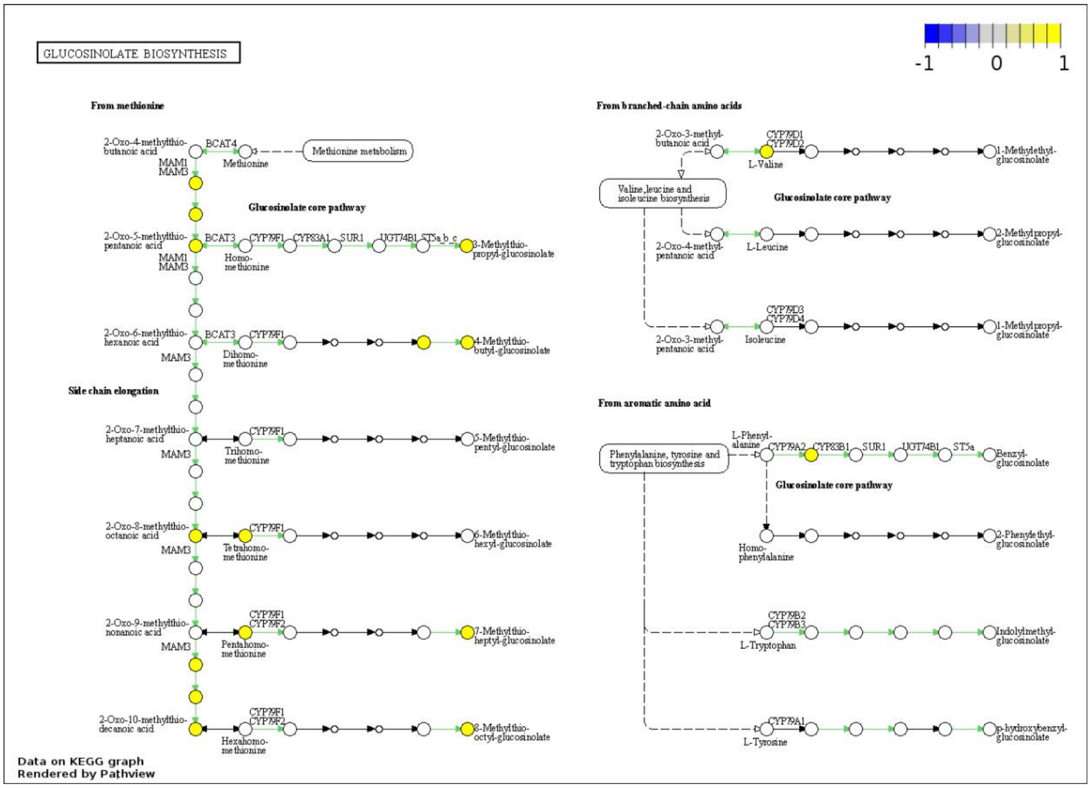
GLUCOSINOLATE BIOSYNTHESIS
-1
0
1
From methionine
2-Oxo-4-methylthio-butanoic acid
BCAT4
Methionine
Methionine metabolism
MAM1
MAM3
Glucosinolate core pathway
2-Oxo-5-methylthio-pentanoic acid
BCAT3
CYP79F1
CYP83A1
SUR1
UGT74B1
ST5a_b_c
3-Methylthio-propyl-glucosinolate
Homo-methionine
MAM1
MAM3
2-Oxo-6-methylthio-hexanoic acid
BCAT3
CYP79F1
4-Methylthio-butyl-glucosinolate
Dihomo-methionine
MAM3
Side chain elongation
2-Oxo-7-methylthio-heptanoic acid
CYP79F1
5-Methylthio-pentyl-glucosinolate
Trihomo-methionine
MAM3
2-Oxo-8-methylthio-octanoic acid
CYP79F1
6-Methylthio-hexyl-glucosinolate
Tetrahomo-methionine
MAM3
2-Oxo-9-methylthio-nonanoic acid
CYP79F1
CYP79F2
7-Methylthio-heptyl-glucosinolate
Pentahomo-methionine
MAM3
2-Oxo-10-methylthio-decanoic acid
CYP79F1
CYP79F2
8-Methylthio-octyl-glucosinolate
Hexahomo-methionine
Data on KEGG graph
Rendered by Pathview
From branched-chain amino acids
2-Oxo-3-methyl-butanoic acid
CYP79D1
CYP79D2
L-Valine
1-Methylethyl-glucosinolate
Valine,leucine and isoleucine biosynthesis
Glucosinolate core pathway
2-Oxo-4-methyl-pentanoic acid
L-Leucine
2-Methylpropyl-glucosinolate
CYP79D3
CYP79D4
2-Oxo-3-methyl-pentanoic acid
Isoleucine
1-Methylpropyl-glucosinolate
From aromatic amino acid
Phenylalanine, tyrosine and tryptophan biosynthesis
L-Phenyl-alanine
CYP79A2
CYP83B1
SUR1
UGT74B1
ST5a
Benzyl-glucosinolate
Glucosinolate core pathway
Homo-phenylalanine
2-Phenylethyl-glucosinolate
CYP79B2
CYP79B3
L-Tryptophan
Indolylmethyl-glucosinolate
CYP79A1
L-Tyrosine
p-hydroxybenzyl-glucosinolate

(e)

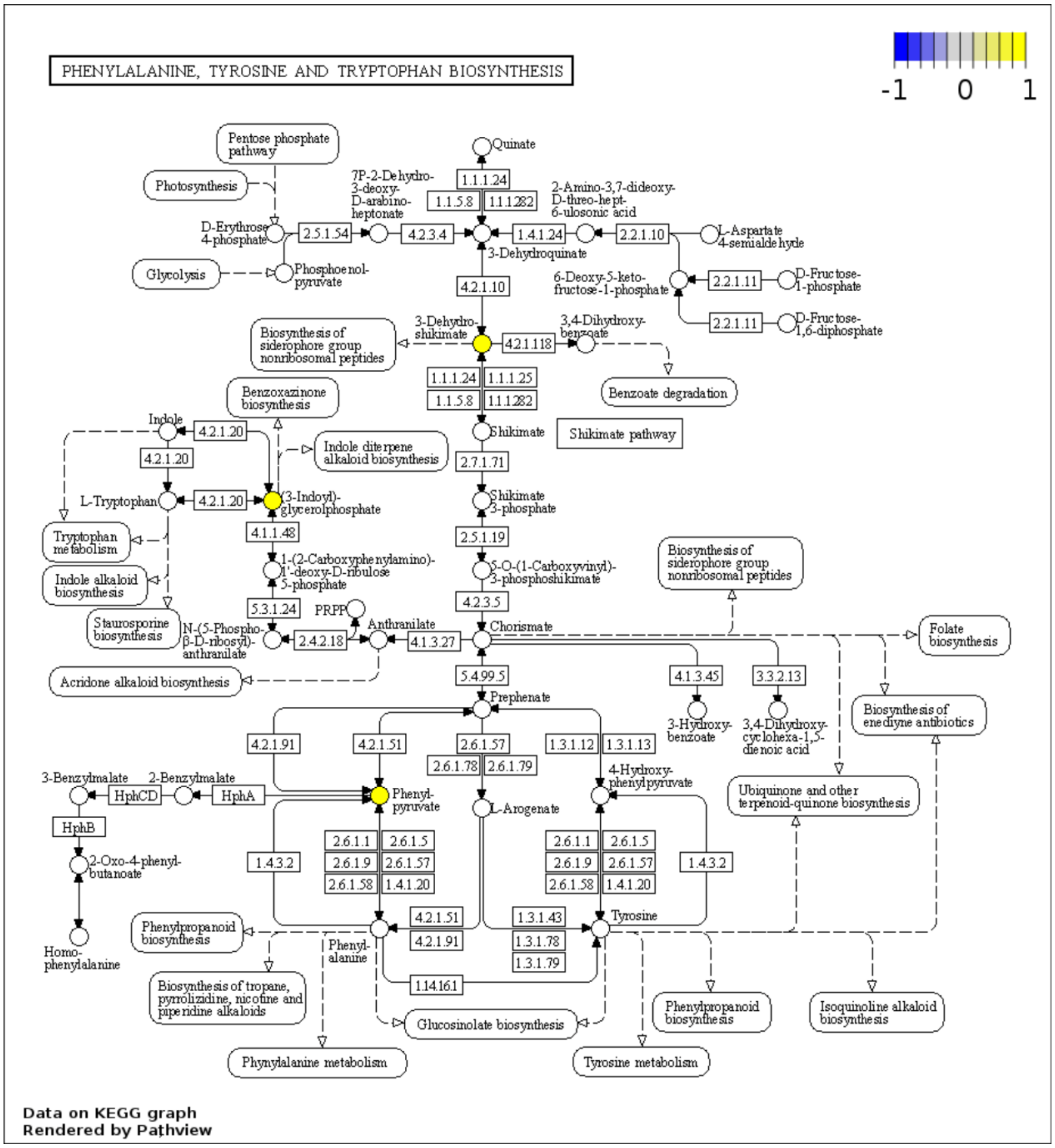
PHENYLALANINE, TYROSINE AND TRYPTOPHAN BIOSYNTHESIS
-1 0 1
Pentose phosphate pathway
Photosynthesis
Glycolysis
D-Erythrose 4-phosphate
Phosphoenol-pyruvate
7P-2-Dehydro-3-deoxy-D-arabino-heptonate
Quinate
3-Dehydroquinate
2-Amino-3,7-dideoxy-D-threo-hept-6-ulosonic acid
L-Aspartate 4-semialdehyde
6-Deoxy-5-keto-fructose-1-phosphate
D-Fructose-1-phosphate
D-Fructose-1,6-diphosphate
3-Dehydro-shikimate
3,4-Dihydroxy-benzoate
Biosynthesis of siderophore group nonribosomal peptides
Benzoate degradation
Benzoxazinone biosynthesis
Indole
Indole diterpene alkaloid biosynthesis
Shikimate
Shikimate pathway
L-Tryptophan
(3-Indoyl)-glycerolphosphate
Shikimate 3-phosphate
Tryptophan metabolism
Indole alkaloid biosynthesis
1-(2-Carboxyphenylamino)-1'-deoxy-D-ribulose 5-phosphate
5-O-(1-Carboxyvinyl)-3-phosphoshikimate
Staurosporine biosynthesis
N-(5-Phospho-β-D-ribosyl)-anthranilate
PRPP
Anthranilate
Chorismate
Folate biosynthesis
Acridone alkaloid biosynthesis
Prephenate
3-Hydroxy-benzoate
3,4-Dihydroxy-cyclohexa-1,5-dienoic acid
Biosynthesis of enediyne antibiotics
3-Benzylmalate
2-Benzylmalate
HphCD
HphA
HphB
Phenyl-pyruvate
L-Arogenate
4-Hydroxy-phenylpyruvate
Ubiquinone and other terpenoid-quinone biosynthesis
2-Oxo-4-phenyl-butanoate
Homo-phenylalanine
Phenylpropanoid biosynthesis
Phenyl-alanine
Tyrosine
Biosynthesis of tropane, pyrrolizidine, nicotine and piperidine alkaloids
Glucosinolate biosynthesis
Phynylalanine metabolism
Tyrosine metabolism
Isoquinoline alkaloid biosynthesis
2.5.1.54
4.2.3.4
1.1.1.24
1.1.5.8
1.1.1.282
1.4.1.24
2.2.1.10
2.2.1.11
4.2.1.10
4.2.1.118
1.1.1.25
4.2.1.20
2.7.1.71
4.1.1.48
2.5.1.19
5.3.1.24
4.2.3.5
2.4.2.18
4.1.3.27
5.4.99.5
4.1.3.45
3.3.2.13
4.2.1.91
4.2.1.51
2.6.1.57
2.6.1.78
2.6.1.79
1.3.1.12
1.3.1.13
1.4.3.2
2.6.1.1
2.6.1.5
2.6.1.9
2.6.1.58
1.4.1.20
1.3.1.43
1.3.1.78
1.3.1.79
1.14.16.1
Data on KEGG graph
Rendered by Pathview

(f)

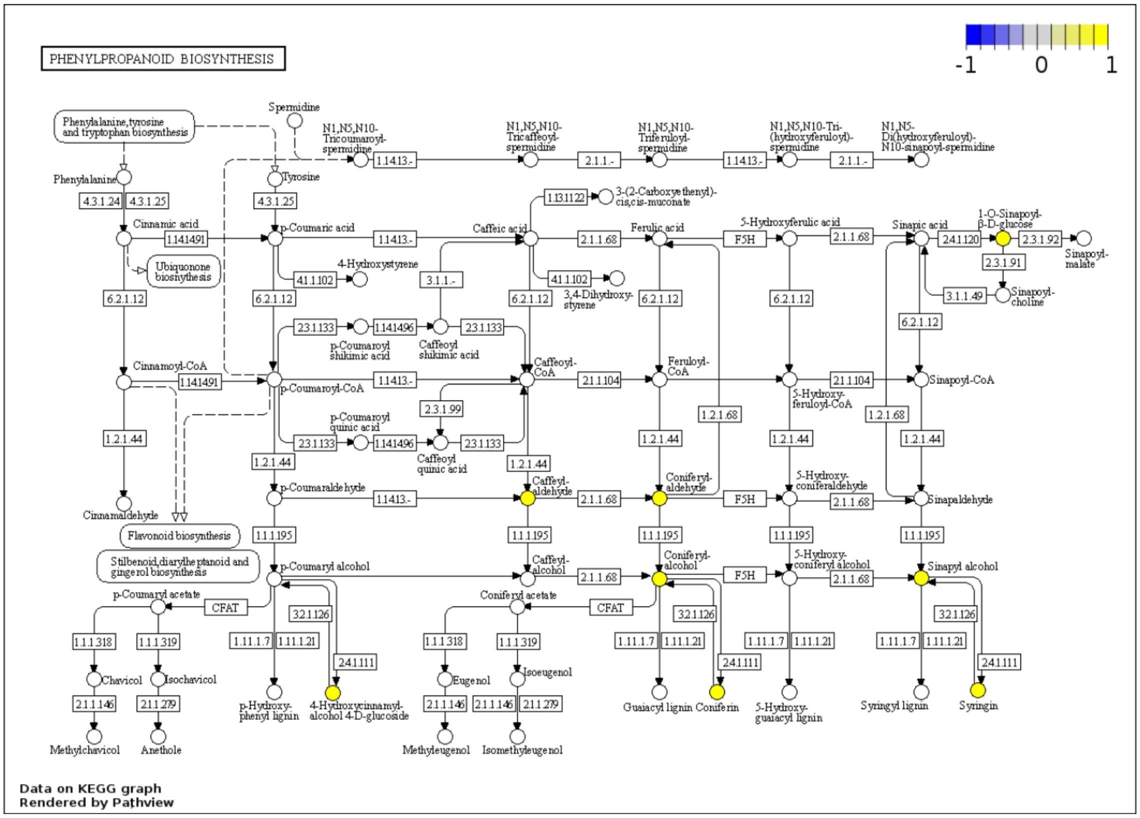

Figure S15. Reconstruction of metabolic pathways of the metabolome of *A. thaliana*. The six main and mostly enriched metabolic pathways corresponding to the secondary metabolism of *A. thaliana* are shown in a representative way. Within each metabolic pathway, the points marked in yellow represent those metabolites identified in mass spectral libraries and with a presumptive annotation by KEGG code. The color scale alludes to the presence/absence of the SMe within the pathway. (**a**) Cutin, suberin and wax biosynthesis, (**b**) flavone and flavonol biosynthesis, (**c**) flavonoid biosynthesis, (**d**) glucosinolate biosynthesis, (**e**) phenylalanine, tyrosine, and tryptophan biosynthesis, (**f**) phenylpropanoid biosynthesis.
